# The Dual Nature of Metals: Essential Nutrients and Environmental Contaminants

**DOI:** 10.3390/ijms27093815

**Published:** 2026-04-25

**Authors:** Marcela Rojas-Lemus, Nelly López-Valdez, Adriana González-Villalva, Patricia Bizarro-Nevares, Brenda Casarrubias-Tabarez, María Eugenia Cervantes-Valencia, Martha Ustarroz-Cano, Norma Rivera-Fernández, Jhony Anacleto-Santos, Fernando Chávez-Maya, Rebeca Milán-Chávez, Sara Morales-López, Teresa I. Fortoul

**Affiliations:** 1 Departamento de Biología Celular y Tisular, Facultad de Medicina, Universidad Nacional Autónoma de México (UNAM), Ciudad de México 04510, Mexico; mrojaslemus@facmed.unam.mx (M.R.-L.); nellylopez@facmed.unam.mx (N.L.-V.); aegonzalez@facmed.unam.mx (A.G.-V.); pbizarro@unam.mx (P.B.-N.); bcasarrubias@facmed.unam.mx (B.C.-T.); maria.cervantes@facmed.unam.mx (M.E.C.-V.); ustarrozcano@hotmail.com (M.U.-C.); 2 Departamento de Microbiología y Parasitología, Facultad de Medicina, Universidad Nacional Autónoma de México (UNAM), Ciudad de México 04510, Mexico; normariv@unam.mx (N.R.-F.); jhony@unam.mx (J.A.-S.); 3 Departamento de Medicina y Zootecnia de Aves, Facultad de Medicina Veterinaria y Zootecnia, Universidad Nacional Autónoma de México (UNAM), Ciudad de México 04510, Mexico; cmayaf@unam.mx; 4 Departamento de Bioquímica, Facultad de Medicina, Universidad Nacional Autónoma de México (UNAM), Ciudad de México 04510, Mexico; milan@bq.unam.mx (R.M.-C.);

**Keywords:** metals, trace metals, toxicity, essentiality

## Abstract

Metals are an essential part of the life of all organisms because they participate as an essential part of diverse components, especially as enzymatic cofactors. In humans, there are metals that are trace elements and therefore are required for the proper functioning of different biological processes, so they must be present in cells and tissues. However, when the organism is overexposed, those same essential metals—in high concentrations that become toxic—cause imbalances or overt pathologies. On the other hand, there are metals that are not essential in humans, so their presence and accumulation in the organism can cause adverse effects. In this review we focus on the essentiality and toxicity of the main trace metals such as iron, zinc, copper, manganese, chromium, cobalt, molybdenum, and nickel, as well as on the toxicity of metals such as vanadium, cadmium, and lead that are not essential for humans. In addition, the report describes the main mechanisms by which metals exert their toxic effects on the body, as well as the primary sources of pollution through which they are released into the environment.

## 1. Introduction

The presence of metals is essential for organisms of the three domains of life (archaeobacteria, prokaryotes, and eukaryotes) because metals participate in key roles within many cellular and survival processes [1]. In 1985, the term trace element or microelement was proposed to refer to elements that are present in very small amounts, below 0.01% of the organism’s weight, but that are vital for cellular function and are considered essential, since their lack causes metabolic and physiological alterations, which can lead to the development of deficiency diseases [2]. In total, twenty elements seem to be essential for the correct functioning of the human body, half of which are metals, and the other half are non-metals. Among the metals that are currently considered essential for normal biological functioning are four elements from the group of alkali and alkaline earth metals: Na, K, Mg, Ca (macroelements), and six transition metal elements: Mn, Fe, Co, Cu, Zn and Mo (microelements) [3]. Trace elements act as enzymatic catalysts (because they are part of their active centers), in addition to being part of the structural and regulatory configuration of multiple hormones and of diverse proteins with and without enzymatic activity [2]. Dysregulation of trace-element homeostasis gives rise to anomalies at the systemic level, which can be due to deficiency, or it can lead to states of toxicity when they accumulate beyond what is necessary [2]. For this reason, cells have developed diverse regulatory mechanisms for metal ions to maintain their homeostasis [3], since there is a large amount of literature showing that trace elements play a fundamental role, both positive and negative, in human carcinogenesis, and that higher levels of some trace metals (such as Mn and Zn) are associated with a higher risk of developing other complications such as hypertension, hyperglycemia, hyperinsulinemia, and dyslipidemia [4]. However, the underlying mechanisms are still not well known [4], so understanding the activities of metals in organisms has become so important that, in 2004, metallomics emerged as the new paradigm regarding metal-biomolecules related to organisms, which takes into account the interactions between toxic and essential metals, transport through biological fluids, passage through membranes and biological interfaces, synergistic and antagonistic actions among metallic species, and alterations in metabolic pathways caused by the overexpression or inhibition of these metal-biomolecules [5]. Because many metals are essential for organisms, but at high doses they can be toxic and carcinogenic [6], in this review we focus on the essentiality and toxicity of the main trace metals such as Fe, Zn, Cu, Mn, chromium (Cr), Mo, and nickel (Ni), as well as on the toxicity of metals such lead (Pb), cadmium (Cd), and vanadium (V) that are not essential for humans. In addition, the main mechanisms of action by which metals exert their toxic effects in the organism are also described.

## 2. Trace Metals: Essentiality and Toxicity

In the human body, trace metals are found in small amounts; it is estimated that Fe is 4 to 5 g, Zn 2 to 3 g, Cu 80–100 mg, Mn 12–20 mg, Cr 6–20 mg, Mo 5–10 mg, and Co 1–2 mg [3]. In this review, nickel is also included, although its essentiality in humans is still debated (as is that of Cr). The characteristics of these metals as essential and toxic elements are described below.

### 2.1. Iron

Iron (Fe, from the Latin *ferrum*) is a transition metal with atomic number 26. It is the most abundant element on Earth, since it is part of both the crust and the core. It is the second most abundant metal in the Earth’s crust, and it has been used since antiquity to manufacture tools, weapons, and machinery, among other applications [7].

#### 2.1.1. Chemical Properties and Metabolism

Fe is found in the Earth’s crust, mainly in two oxidation states: Fe(III), or ferric, its oxidized state, and Fe(II), or ferrous, its reduced and more soluble state [8].

Intestinal absorption is the primary control point for this metal’s entry into the body. Excretion, on the other hand, occurs through passive pathways or those associated with other physiological processes, such as cell turnover, sweating, or menstruation, rather than through a homeostatic control system like that of absorption [9]. Inorganic iron enters through transporter proteins, such as DMT1 (Divalent Metal Transporter 1), expressed in duodenal enterocytes. Subsequently, ferroportin, located on the basolateral membrane of these cells, transports iron into the systemic circulation. While ferroportin is primarily expressed in the duodenal mucosa, it is also present in hepatocytes and macrophages [10]. Once absorbed, it can bind to transferrin to be transported from plasma to tissues, and it enters cells through the transferrin receptor via endocytosis, especially in hepatocytes, macrophages, and bone marrow cells. The excretion mechanism is the loss of iron through the desquamation of epithelial cells, especially from the skin and hair, and through the rapid turnover of enterocytes [9].

Iron is mainly found in erythrocytes, in the heme group of hemoglobin and, to a lesser extent, in muscle myoglobin, as well as being concentrated in storage proteins such as ferritin and hemosiderin. It is also found to a lesser extent in proteins and enzymes, such as the cytochromes of the respiratory chain, cytochromes p450, and some redox enzymes [9].

An important mechanism for regulating iron levels in the blood is hepcidin, a hormone produced by the liver that binds to ferroportin, stimulating its endocytosis and degradation. This reduces the release of iron by the liver and macrophages, as well as iron absorption in the intestine [7]. When iron stores decrease, hepcidin production is inhibited to increase iron absorption. An increase in hepcidin can lead to iron overload, and a decrease can lead to iron deficiency. The hepcidin/transferrin system is very important for understanding both iron deficiency and iron overload in the body [10].

#### 2.1.2. Sources of Iron

The main source of Fe is dietary intake, including meat, legumes, and green leafy vegetables. WHO recommends 60 mg of elemental iron per day. The recommended daily intake of Fe depends on the life period: for pregnant women it is 27 mg, for premenopausal women it is 18 mg, for men it is 8 mg, and for children 7 to 12 months of age it is 11 mg. Normal serum iron levels are 55–176 µg/dL in men and 40–170 µg/dL in women [11]. Another source of exposure is through the presence of atmospheric Fe. For example, one of the sites with the highest concentration of iron in suspended particles is collective subway transportation [12], since wear of the rails and train wheels generates particles that are easy to inhale and are made up of iron or of Fe–Mn or Fe–Cr alloys (alloys used in the manufacture of the wheels) [13]. Another source of Fe is water. In studies of water reservoirs near abandoned coal mines, iron levels above the limits established by the WHO have been detected. Pyrite mineral deposits and tools used in mines, when they oxidize, release Fe ions. This process is also increased by the metabolic activity of certain microorganisms [14].

#### 2.1.3. Biological Functions of Iron

Fe is essential because it is a cofactor in various chemical reactions, in oxygen transport, as well as in DNA synthesis and metabolism. Six-point five percent of all enzymes in the organism require iron. Its functions depend on its oxidation state: ferrous (+2), ferric (+3), and ferryl (+4). It has a great ability to change its oxidizing potential, its redox potential, and its oxidation state, which explains its participation in a wide variety of chemical reactions. The main ligands of Fe are nitric oxide, oxygen, and sulfides. Proteins that require iron include hemoglobin, myoglobin, and cytochromes of the respiratory chain, among others. One of the main functions of iron is oxygen transport to the mitochondria [15].

#### 2.1.4. Iron Deficiency

Fe deficiency is more prevalent in women and children. Worldwide, 30% of women and 40% of pregnant women and children under 5 years of age have iron deficiency. The most common causes of Fe deficiency are poor nutrition in young children and chronic blood loss in adolescents and adults. Rarer causes are malabsorption syndromes and some rare genetic factors that affect iron metabolism [10].

Fe deficiency causes inefficient hemoglobin production and disrupts erythrocyte formation. Erythrocytes are usually smaller and paler, which leads to a hypochromic microcytic anemia [7]. It is important to mention that this type of anemia is not only due to Fe deficiency; it can also be due to other causes that affect hemoglobin production. Patients usually present pallor and fatigue, but they may also present cheilitis, atrophic glossitis, and pica (the desire to eat non-edible objects, for example, soil). Neurocognitive and behavioral problems have also been described in both children and adults [10].

Due to the importance of Fe in oxygen transport and its function in the mitochondria of cardiac muscle, its deficiency is associated with heart failure exacerbated by anemia [15]. A problem associated with iron deficiency is that several metals share the absorption pathway. Thus, in deficiency of this metal, transporters such as DMT1 increase; in addition to Fe, it can transport other metals, such as lead [16].

#### 2.1.5. Iron Intoxication

Iron overload is associated with hyperabsorption of iron or with its parenteral administration, including blood transfusion, rather than impaired excretion, which is normally so limited. Genetic alterations of Fe metabolism, such as hemochromatosis, cause the accumulation of this metal mainly in hepatocytes. Hemosiderosis is also an Fe overload, but accumulation occurs in liver macrophages and may be due to ineffective erythropoiesis, to hemolytic anemias that increase intestinal iron absorption and require frequent blood transfusions [10]. Fe overload is diagnosed more frequently in men, since in the reproductive stage menstrual losses act as a protective factor in women. Its effects include liver damage, inflammation, fibrosis, and, if not diagnosed in time, liver cirrhosis. Treatment consists of phlebotomies to remove excess iron or administering chelating agents. An increased risk of liver cancer, diabetes mellitus, and cardiomyopathies has been reported in these patients [9].

If transferrin saturation exceeds 40%, it supports the diagnosis of iron overload [10], but if it increases to 60–70%, the amount of free iron is much higher and it is more likely to damage tissues, accumulate in the liver and in cardiac muscle cells, and cause oxidative-stress damage. In the liver it can lead to fibrosis, cirrhosis, and an increased risk of liver cancer. In the heart it predisposes to arrhythmias and heart failure. Endocrine glands are also susceptible to iron damage, which can lead to diabetes, hypothyroidism, and hypogonadism in some cases [10].

Death from fulminant hepatic failure after acute ingestion of large amounts of Fe has been reported in a human case and was subsequently studied in a mouse model [17]. Chronic ingestion of iron supplements has also caused increased ferritin and liver damage, although not as severe as that caused by hemochromatosis due to genetic problems [9].

The review by Teschke et al. describes various studies addressing the potential toxic effects of iron; however, it is essential to distinguish the level of evidence: on the one hand, there are animal models (such as studies in rats) that have evaluated mechanisms of intestinal iron absorption and hepatic overload, and in vitro studies that have explored pathways such as ferroptosis and iron-induced oxidative stress. However, evidence in humans is limited or inconsistent: although there are reports of acute iron poisoning from intentional or accidental ingestion leading to fulminant liver failure (case studies), as well as a case of chronic overload from prolonged use of oral supplements, epidemiological studies have failed to convincingly demonstrate that environmental exposure to iron (for example, in steel mill or mine workers) causes clinically relevant liver damage. In fact, the text itself notes that most claims regarding health risks from environmental iron lack solid evidence and that healthy individuals without genetic mutations possess homeostatic mechanisms that limit intestinal absorption of excess iron. Therefore, although experimental and observational studies exist, the evidence of significant liver toxicity from iron in humans exposed to environmental sources is weak and inconclusive [9].

**Figure 1:**
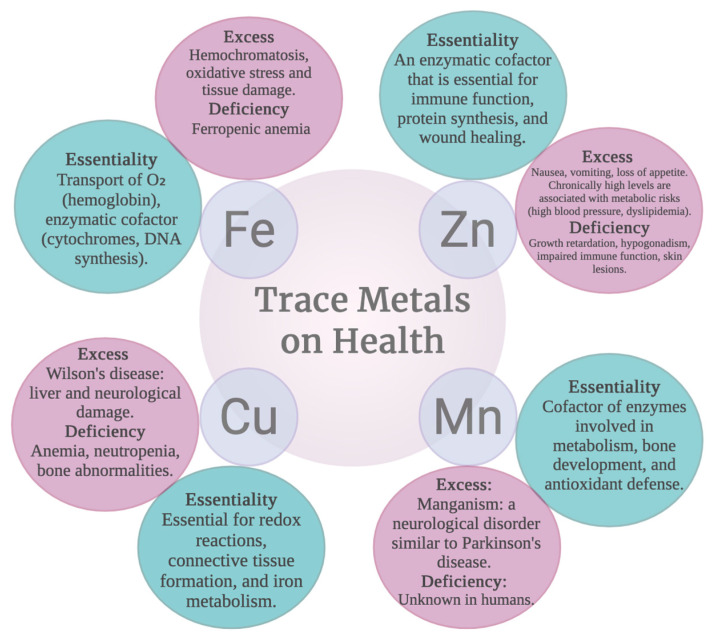
Summary of the essentiality, excess, and deficiency of the trace metals Fe, Zn, Cu, and Mn. Created in https://BioRender.com.

Among the toxic effects are associations with neurodegenerative diseases, cognitive impairments, and pulmonary problems, among others. In a cohort study, a strong association was found between Fe and Cu concentrations in suspended PM2.5 particles, the generation of reactive oxygen species, and increased risk of cardiovascular diseases [18]. There are also some iron compounds in asbestos fibers, in cigarettes, or that form when grilling red meat, which have been associated with a higher risk of cancer [19]. Figure 1 summarizes the toxic and essential effects of Fe.

### 2.2. Zinc

Its name comes from the German word zinke, which translates as point [20]. Zn was identified as an essential element for all living beings in 1869 based on findings in Aspergillus niger; in 1933 it was shown to be essential in plants and for normal development of rats and birds [21].

#### 2.2.1. Chemical Properties and Metabolism

Zinc is in group 12 of the periodic table and its atomic number is 30; it is a white metal with bluish tints, shiny, brittle when cold, and at temperatures between 100 and 150 °C it becomes malleable and ductile [20]. It is the most abundant trace element after iron. It is found as a divalent cation (Zn^2+^), and under physiological conditions it does not have an active redox effect. For its cellular uptake different types of membrane transporters are required, whose presence is necessary for homeostasis of this metal [22]. Zn utilizes two families of widely expressed and finely regulated transporters: ZnT (SLC30A) and ZIP (SLC39A). These systems are essential not only for Zn homeostasis but also for cellular signaling and the prevention of Zn toxicity [20]:

- ZIP Family (Zrt-, Irt-like Proteins; SLC39A): These transporters increase cytosolic Zn concentration by mobilizing the ion from the extracellular space or from within organelles into the cytosol. In the liver, members such as ZIP14 (SLC39A14) and ZIP8 (SLC39A8) are particularly relevant, as they mediate Zn uptake from the portal blood into hepatocytes. Furthermore, these transporters have relatively broad specificity and can transport other toxic metals such as cadmium (Cd^2+^), Mn, and non-transferrin-bound Fe (NTBI). Overexpression of ZIP14 during the acute-phase response (induced by IL-6) contributes to hypozincemia and may alter hepatic uptake of other heavy metals.
- ZnT Family (Zinc Transporters; SLC30A): They act in the opposite direction to ZIPs, exporting Zn from the cytosol to the outside of the cell or into organelles (Golgi apparatus, synaptic vesicles, secretory granules). In the liver, ZnT1 (SLC30A1), located on the basolateral membrane of hepatocytes and cholangiocytes, is crucial for exporting Zn into the blood or bile, protecting the cell from toxic overload. ZnT7 and ZnT5 are important for loading Zn into the endoplasmic reticulum and the Golgi apparatus, activating zinc-dependent enzymes such as alkaline phosphatases.

#### 2.2.2. Sources of Zinc

Dietary intake is the main source of this element. The foods that provide the most Zn are red meats and seafood, followed by cereals, nuts, and legumes. In addition, over-the-counter dietary supplements often contain zinc as part of their components [22].

The amount of Zn that an organism can absorb varies according to diet; for example, in a vegetarian diet, the large amount of phytate it contains causes a negative effect on Zn bioavailability. Diets that include seafood and meat are rich in Zn, since they have low phytate content. This suggests that people with vegan or vegetarian diets may have Zn deficiency. The recommended daily consumption is 11 mg/day for men and 8 mg/day for women [23]. Zinc pollution stems primarily from industrial activities such as mining, metal smelting (steel), galvanizing, waste incineration, and the combustion of fossil fuels. It is also released through sewage sludge, fertilizers, pesticides, and the leaching of construction materials; as a result, soil, air, and water can all become contaminated with excessive levels of zinc [24].

#### 2.2.3. Biological Functions of Zinc

This element is essential for the proper functioning of several metalloenzymes, such as alcohol dehydrogenase, alkaline phosphatase, carbonic anhydrase, aminopeptidase, superoxide dismutase, DNA deoxypolymerase, and RNA deoxyribonuclease. It is also required for the metabolism of nucleic acids, membrane proteins, as well as for cell growth and division; it plays a relevant role in maintaining the structure of nucleic acids of genes (zinc fingers). It plays a relevant role in collagen synthesis, bone mineralization and turnover. They act in the insulin signaling pathway to stimulate lipogenesis and glucose uptake by adipose tissue. It also participates in thyroid hormone metabolism by regulating the production of releasing factor (TRF) and thyroid-stimulating hormone (TSH). It is essential in male fertility, since a Zn-sensing receptor has been found in the sperm tail and in the acrosome. When extracellular Zn binds to this receptor, it triggers signaling pathways that increase sperm motility and acrosome exocytosis. It also participates in the development and function of the central nervous system, since its balance is very important in neural tube formation and stem-cell proliferation during development. Several Zn-dependent enzymes also modulate various postsynaptic receptors [21,22,25,26].

It has been estimated that one in ten proteins in the organism is a Zn protein, and more than 300 enzymes and 1000 transcription factors depend on the presence of Zn [22].

The human body contains between 2 and 3 g of Zn. Approximately 60% is stored in skeletal muscle, 30% in bone, 5% in the liver, and the rest in other tissues. Notably, the highest concentrations of Zn are found in the retina and in the vascular layer of this organ [25]. In plasma, most Zn is bound to albumin. Intestinal zinc absorption (in the duodenum and jejunum) is highly regulated; when there is a deficiency, absorption in these organs increases up to 90%. Excess is eliminated through desquamation of cells of the digestive tract and skin, together with renal excretion. Within cells, 50% of zinc is in the cytoplasm, 30 to 40% within the nucleus, and the rest in the plasma membrane [21]. From 5 to 15% of cytoplasmic Zn is bound to metallothioneins that serve as Zn donors and acceptors, in addition to transporting it. Zn transporters (ZnT) move it from the cytosol to the extracellular space and intracellular spaces, while ZIP transporters have the function of replenishing cytoplasmic Zn from that located in the extracellular space and in intracellular compartments. Each transporter plays a different role in Zn transport. Most ZIP transporters are in the cell membrane and increase when there is a Zn deficiency [21].

There are organs that accumulate more zinc than others [26]. The accumulation of zinc in tissues is not a random phenomenon, but rather reflects specialized physiological functions. For example, the prostate has the highest concentrations of zinc in soft tissues, which is essential for inhibiting mitochondrial aconitase and maintaining citrate production in prostatic fluid—a process that is lost in prostate cancer. Similarly, the secretory granules of specialized cells such as mast cells, intestinal Paneth cells, and adenohypophyseal cells store zinc for regulated release, acting as a second messenger in immunity (mast cells), in the host’s defense against pathogens (Paneth cells), or in hormonal secretion, respectively. Thus, the local accumulation of zinc in specific tissues and organelles is a critical requirement for highly specialized signaling, defense, and energy metabolism processes [25]. Zn absorption can be altered by the presence of other elements such as Cu and Fe and one way to regulate the amount of Zn in the organism is through chelation [23,27].

#### 2.2.4. Zinc Deficiency

People with a deficiency of this microelement may present diarrhea, impaired immune function, infections, memory loss, cognitive alterations, and sperm damage in men. Populations at risk of Zn deficiency are pregnant individuals, infants, users of vegetarian and vegan diets, people over 75 years old, users of proton pump inhibitor drugs, tetracyclines and quinolones, as well as children with sickle cell anemia, patients with anorexia nervosa, or with Crohn’s disease. The manifestations of Zn deficiency are very variable; among them are growth retardation, hypogonadism, osteopenia, weight loss, depression, difficulty concentrating, nystagmus, dysarthria, night blindness, hypogeusia, anosmia, alopecia, dermatitis, paronychia and stomatitis, diarrhea, glossitis, pica, anorexia, fever, infertility, frequent infections, alteration in glucose tolerance, and problems during pregnancy and dementia are reported alterations [25].

#### 2.2.5. Zinc Intoxication

Above 40 mg/day, zinc is considered potentially toxic when administered orally [22]. Individuals can suffer intoxication due to excess Zn in various circumstances, including excessive consumption of Zn supplementation, patients with Wilson’s disease (Cu accumulation), workers in industries related to Zn smelting and mining [22,23], and excessive use of adhesives for dental prostheses [26].

Data that may suggest chronic zinc intoxication are alterations associated with copper deficiency that would produce alterations in the immune system, a decrease in high-density lipoproteins (HDLs) and an increase in low-density lipoproteins (LDLs), systemic arterial hypertension. Elevation in Zn concentration is associated with neurodegenerative diseases such as Alzheimer’s and Parkinson’s, as well as with multiple sclerosis, together with increased risk of developing prostate cancer [23]. Figure 1 summarizes the toxic and essential effects of Zn.

### 2.3. Copper

Copper (in Latin, *cuprum*) is a transition metal whose interaction with humans dates back to prehistory. Its early use was driven by its favorable physical properties, highlighting its ductility and corrosion resistance [28].

#### 2.3.1. Chemical Properties and Metabolism

At the biological level, Cu is an essential trace element, indispensable as an enzymatic cofactor in multiple metabolic pathways. The functionality of Cu in biological systems derives from its ability to alternate between its oxidation states Cu(I) and Cu(II), which positions it as a determining redox cofactor [29]. This metal exhibits high solubility after ingestion, being able to reach up to 80% solubility in vitro in the acidic environment of the stomach [30]. Cu requirements vary according to life stage and physiological state. For children (0 months to 13 years), the adequate daily intake ranges from 340–700 µg/day; for adults (19–50 years), it is 900 µg/day. These needs increase during pregnancy and lactation, requiring between 1000–1300 µg/day. If oral supplementation is required, copper gluconate is a form recommended by the U.S. Pharmacopeia [31]. Regulation of Cu homeostasis is a process of molecular precision [29,30,32], in which absorption is carried out in the intestine, and it is transported to the portal circulation toward the liver by the ATP7A protein. The liver is the main organ of accumulation, where homeostasis is controlled by ceruloplasmin and the copper pump ATP7B. ATP7B is crucial, since it facilitates both the incorporation of Cu into vital metalloenzymes (such as cytochrome c oxidase) and the biliary (endogenous) excretion of excess Cu bound to metallothionein [33].

#### 2.3.2. Sources of Copper

The general population is exposed to copper daily through inhalation of ambient air, ingestion of food, and drinking water—some of which contains copper, though not all. To a lesser extent, exposure also occurs through skin contact with copper-containing materials [34].

Primary dietary sources of Cu include foods such as organ meats, seafood, nuts, and seeds [35]. Copper pollution comes mainly from mining activities, smelters, manufacturing industries, the use of pesticides and fungicides, and corrosion of household plumbing. These sources include copper-rich dust, industrial wastewater, and sludge. In nature, copper is released through rock erosion, wildfires, and volcanic eruptions [34].

#### 2.3.3. Biological Functions of Copper

It is essential for hemoglobin synthesis, iron oxidation, cellular respiration, amidation of antioxidant peptides, formation of pigments and connective tissue. Copper deficiency affects vital physiological systems such as bone marrow hematopoiesis (especially erythropoiesis), optic nerve function, and the nervous system in general. This is because copper-containing enzymes perform essential functions in the body, including [36]:

Hemoglobin synthesis and iron oxidation, since Cu is an essential cofactor for ceruloplasmin and hefestin. Both are ferroxidases (enzymes that oxidize iron from Fe^2+^ to Fe^3+^). This oxidation is necessary for iron to be loaded onto its transport protein (transferrin) and reach the bone marrow to be incorporated into hemoglobin. Without Cu, Fe accumulates in tissues (liver, pancreas) and cannot be used to form red blood cells, causing microcytic anemia that does not respond to iron supplementation.

*Cellular respiration (ATP production)*: Cu is an essential cofactor of cytochrome c oxidase (Complex IV) in the mitochondrial respiratory chain. This enzyme transfers electrons to molecular oxygen, the final step of aerobic respiration.

*Antioxidant defense*: Cu is a cofactor of superoxide dismutase (SOD1), located in the cytoplasm. SOD1 catalyzes the dismutation of the superoxide radical (O_2_^−^) into hydrogen peroxide (H_2_O_2_) and oxygen, protecting cells from oxidative damage.

*Connective tissue formation (collagen and elastin)*: Cu is a cofactor for lysyl oxidase, which is the enzyme that oxidizes lysine residues in collagen and elastin, allowing the formation of cross-links that provide strength and elasticity to tissues. Its deficiency explains problems such as skin laxity, hernias, or aneurysms (rare in acquired deficiency, but typical of copper deficiency in Menkes syndrome).

*Neurotransmission (biosynthesis and modulation)*: Cu is a cofactor for dopamine β-hydroxylase (converts dopamine to norepinephrine) and monoamine oxidase (MAO) (degrades neurotransmitters such as serotonin and dopamine). Copper deficiency disrupts the balance of catecholamines in the nervous system, contributing to neurological symptoms.

#### 2.3.4. Copper Deficiency

Imbalance in Cu concentration manifests in various pathologies [35]. For example, short-term copper deficiency in neonates has been related to abnormalities in connective tissue, pulmonary insufficiency, neuronal degeneration, bone defects, and a lower survival rate. Prolonged Cu deprivation is associated with ultrastructural cardiac abnormalities, suppression of the immune system, and deterioration of cognitive and behavioral functions. Menkes disease causes a severe copper deficiency (accumulation in tissues, deficiency in the brain) and leads to neurodegeneration [37].

Cardiac defects are caused by decreased lysyl oxidase (LOX) activity and altered mitochondrial ultrastructure, since copper deficiency severely reduces the activity of LOX, a copper-dependent enzyme critical for the cross-linking of collagen and elastin; on the other hand, it causes mitochondrial dysfunction and oxidative stress, because Cu deficiency affects cytochrome c oxidase (COX, Complex IV), a copper-dependent mitochondrial enzyme essential for oxidative phosphorylation. Reduced activity limits ATP production and is associated with mitochondrial damage. Neurological defects are attributed to reduced activity of key copper-dependent enzymes in the brain (COX, DBH) and impaired myelination, because prenatal Cu deficiency causes a marked reduction in COX activity in motor neurons, leading to myelin aplasia and irreversible neurological symptoms [35]. Furthermore, Cu deficiency causes alterations in the catecholaminergic system, as it reduces the activity of dopamine-β-hydroxylase (DBH), a copper-dependent enzyme that converts dopamine into norepinephrine [37]. Defects in connective tissue formation are related to decreased activity of LOX and its homologs (LOXL1-4), which impedes the normal cross-linking of collagen and elastin. In Cu deficiency, LOX activity is drastically reduced because it requires this metal for the self-generation of its cofactor, lysine tyrosylquinone (LTQ); without Cu, this process fails, and the enzyme becomes inactive [37]. In Menkes syndrome, the hair is twisted, a condition known as “pili torti,” due to defects in keratin cross-linking. In animal models, it has been observed that Cu deficiency during gestation leads to aortic aneurysms, subcutaneous hemorrhages, and wound dehiscence, all of which are due to the fragility of connective tissue [35,37]. Taken as a whole, these mechanisms explain why the manifestations of Cu deficiency are systemic and, often, irreversible if they occur during fetal or neonatal development.

#### 2.3.5. Copper Intoxication

Excess copper in the body, whether due to genetic causes such as Wilson’s disease (mutations in the ATP7B transporter) or exposure to copper ionophores, triggers severe cellular toxicity characterized by oxidative stress, mitochondrial damage (including loss of iron-sulfur clusters and inhibition of the respiratory chain), and a specific form of programmed cell death called cuproptosis, in which copper binds to lipoylated proteins of the Krebs cycle in a manner dependent on the FDX1 enzyme. Clinically, this manifests as liver disease (steatosis, cirrhosis, failure) and neurological disease (tremors, Parkinsonism, ataxia). Treatment includes copper chelators (D-penicillamine, trientine), zinc to reduce copper absorption, and, in severe cases, liver transplantation or emerging therapies such as AAV-ATP7B gene therapy [37]. Figure 1 summarizes the toxic and essential effects of Cu.

### 2.4. Manganese

In 1837, Scottish physician John Couper documented the first case of manganese neurotoxicity among workers at a bleaching factory in Glasgow, who exhibited weakness in the legs, unsteady gait, a blank expression, and difficulty speaking. Couper correctly identified the inhalation of manganese oxide dust as the cause, predating other reports by decades. This finding is significant because manganese—essential for producing chlorine and later a key component of steel—extended occupational risks to industries such as welding and battery manufacturing [38]. Due to its chemical characteristics, Mn is widely used in the metallurgical industry, such as in the production of stainless steel and in aluminum alloys [39].

#### 2.4.1. Chemical Properties and Metabolism

Manganese (Mn) is a transition metal whose toxicological significance stems primarily from its ability to exist in multiple oxidation states, particularly Mn^2+^ and Mn^3+^. The latter is the most biologically relevant state, as it can cross biological barriers, accumulate in the basal ganglia, and trigger oxidative stress, mitochondrial dysfunction, and neuroinflammation—mechanisms central to chronic manganese neurotoxicity (manganism) [38].

Because Mn is naturally found in foods, Mn enters the organism orally and is absorbed mainly in the intestine; it enters cells via divalent metal transporters (DMT1 and ZIP8) [40]. Once in the bloodstream, it binds to transport proteins such as transferrin or circulates as a free ion (Mn^2+^) and accumulates in organs such as the brain and the liver [41]. Regulation of systemic Mn concentration is essential to maintain homeostasis, since both deficiency and excess are adverse conditions for the organism: in recent years, the enzyme PHD2 has been identified as the intracellular Mn sensor that initiates the homeostatic response against excess Mn by activating the HIF pathway to increase expression of the exporter SLC30A10 through which this metal is eliminated [42]. When this sensor fails, the consequence is toxic Mn accumulation. Mn is excreted mainly via the biliary route, as part of feces.

#### 2.4.2. Sources of Manganese

The main sources of Mn are foods rich in this element (such as whole grains, rice, nuts, seafood, oats, tea, and multivitamins) and drinking water [40,43]. Mn is present in significant amounts in infant and neonatal formulas: these solutions contain many essential trace elements for survival, but they can also lead to toxic accumulation when administered for prolonged periods or when there is dysregulation in the homeostatic Mn absorption–excretion mechanism [39].

On the other hand, Mn is present in widely used fungicides (maneb and mancozeb), in fuel additives, and in the gasoline additive MMT (methylcyclopentadienyl manganese tricarbonyl), in addition to being emitted into the atmosphere during mining activity and battery manufacturing [43]. Inhalation exposure is the route by which this metal is absorbed best and most, and it is also the route of greatest concern in terms of health, since the metal that enters by this route bypasses toxicity control processes by the intestinal barrier and by hepatic activity [43].

#### 2.4.3. Biological Functions of Manganese

Manganese is an essential metal because it is necessary for the proper functioning of the immune system, regulation of blood sugar levels and cellular energy, reproduction, digestion, bone growth, blood clotting, hemostasis, and defense against reactive oxygen species. The beneficial effects of manganese are due to the incorporation of the metal into metalloproteins. The functions performed by manganese metalloproteins include oxidoreductases, transferases, hydrolases, lyases, isomerases, and ligases. In addition, manganese is incorporated into the arginase enzymes, glutamine synthetase, phosphoenolpyruvate decarboxylase, pyruvate carboxylase, and manganese superoxide dismutase [40,44]. Mn is one of the most common metals in mammalian tissues.

Daily Mn requirements (according to different health institutions such as the FDA) differ across age groups; for example: adult men 2 mg/day; adult women 1.8 mg/day; children 4–8 years 1.5 mg/day; children 7–12 months 600 µg/day [45].

#### 2.4.4. Manganese Deficiency

Mn deficiency is uncommon, because it is present in numerous dietary sources, and experimentally, the effect of its deficiency has not been described. However, inadequate intake causes deficient growth, poor bone formation and skeletal defects, abnormalities in glucose tolerance, and alteration of lipid and carbohydrate metabolism [39]. Experimentally, men have been placed on low-Mn diets, and it was observed that they developed transient skin eruptions, as well as decreased serum cholesterol and effects on bone remodeling. It has also been shown that insufficient Mn concentration affects reproductive development, alters mood states, increases premenstrual pain in women of reproductive age, and, in children, has been associated with lower scores on tests of cognitive flexibility and speed of thinking [39] (Figure 1).

#### 2.4.5. Manganese Intoxication

Mn intoxication in humans is increasingly frequent due to high Mn levels in contaminated water and inhalation exposure to fumes that contain it; in addition, injection of intravenous drugs has caused an increase in neurological disorders directly associated with this metal [40]. However, excess Mn causes serious harmful effects on human health. These effects are observed especially in the central nervous system, since Mn accumulates in the brain [46].

Mn accumulates especially in the cerebral basal ganglia and generates a condition clinically like Parkinson’s disease known as manganism [40], which was initially observed in occupationally exposed personnel working in mining, metallurgy, battery production, and ferromanganese alloy plants. However, environmental exposure to this metal has gained importance and has become a public health problem, especially concerning susceptible populations such as children [47]. Studies show that high exposure is associated with reduced IQ, increased oxidative stress, mitochondrial dysfunction, endoplasmic reticulum stress, increased cell death, neuroinflammation, and interference with neurotransmitter metabolism; moreover, in recent studies it has been shown that Mn produces alterations in epigenetic regulation, which suggests that it affects cellular programming, and that these alterations can affect neurological development and vulnerability to developing other nervous system disorders [46]. Figure 1 summarizes the toxic and essential effects of Mn.

### 2.5. Chromium

The name Cr derives from the Greek word “chrōma” (χρώμα), which means color [48]. It is not until the early 20th century that the use of Cr in industry increases to make alloys due to its high resistance to heat and corrosion [49].

#### 2.5.1. Chemical Properties and Metabolism

Cr is a transition metal with oxidation states ranging from −2 to +6. The most stable and common are +2, +3, and +6. The +3 state is usually the most stable; +4 and +5 are rare, and the +6 state, or hexavalent chromium (Cr(VI)), is highly toxic and carcinogenic [48,49].

The oxidation state of Cr determines its metabolism and toxicity; for example, Cr(III) is relatively stable, is poorly absorbed, is of low toxicity, and is rapidly eliminated in urine, is not a strong oxidant, and reacts slowly to form complexes; whereas Cr(VI) is a strong oxidant, rapidly forms complexes, oral or inhalation absorption is very high, it is highly toxic, it accumulates in organs, and it is eliminated in urine upon being reduced to Cr(III) [49].

#### 2.5.2. Sources of Chromium

Inhalation exposure represents the greatest risk, especially at the occupational level where personnel are exposed to Cr vapors or dust in the air. Other routes of exposure are oral, through consumption of contaminated water, and dermal, through skin exposure to Cr dust and aerosols [49].

Chromium (Cr) is a metal of wide use in metallurgical and chemical industries; therefore, fumes from industries related to electroplating and welding are important sources of exposure. Cr is also used in the manufacture of dyes, leather preparation, metal smelting, and the manufacture of catalysts to process hydrocarbons; these types of industries can generate waste that contaminates air, water, and soil, being important sources of occupational exposure and for the general population. In addition to the above, it is of interest to highlight that tobacco smoke is also a source of exposure to Cr [49].

Diet is another source of chromium; there are some foods that contain this element naturally. However, this element is ubiquitous in foods at very low concentrations, since most of the chromium they contain and, therefore, in the diet, derives from food processing with stainless steel equipment; therefore, it is likely that humans evolved from a diet that contained much less chromium than what it contains today [50].

#### 2.5.3. Biological Functions of Chromium

There is controversy surrounding the health benefits of chromium, as it is still an ambiguous essential mineral; it is mentioned that Cr(VI) has no verified biological function and has been classified as nonessential for mammals. However, it is common for Cr(III) to be added to dietary supplements, and it has been suggested that its moderate intake poses no risk and is even considered a trace element [48]. It is found naturally in various foods including egg yolk, bran, coffee, nuts, broccoli, meat, and brewer’s yeast, among others [51]. The recommended maximum daily intake of chromium is 10 to 40 µg for children up to six months, 35 µg in men, and 25 µg in women. Cr(III) is attributed antidiabetic activities because it is a component of the glucose tolerance factor (GTF), which, upon binding to insulin, enhances its action and in response decreases blood glucose concentrations [48,51].

#### 2.5.4. Chromium Deficiency

Cr can exist in several oxidation states, with trivalent chromium (Cr^3+^) being the biologically relevant form found in foods. Hexavalent chromium (Cr^6+^) is toxic and has no nutritional value.

The role of Cr(III) in human nutrition and glucose metabolism is highly controversial. In the past, it was proposed that chromium forms an integral part of a hypothetical “glucose tolerance factor” (GTF) that facilitates insulin action. However, despite decades of research, this complex has never been isolated or structurally characterized, and the existence of a specific GTF is no longer accepted by the scientific community [51].

While some observational studies have reported lower serum Cr levels in patients with type 2 diabetes (T2DM) compared to healthy controls, the clinical significance of this finding remains uncertain. Large-scale intervention trials and meta-analyses have shown inconsistent and, at best, modest effects of chromium supplementation (e.g., chromium picolinate) on glycemic control markers such as glucose or HbA1c. Furthermore, a causal link between chromium deficiency and the pathogenesis of T2DM has not been established. Recent research suggests that any association between Cr status and T2DM may be confounded by other variables and does not persist after rigorous statistical correction, in contrast to the robust and well-defined role of elements like zinc [52].

Therefore, while Cr is an essential trace element for some animals, its essentiality in humans has not been definitively proven. Its use as a dietary supplement for improving glucose metabolism remains a subject of debate, and its effects, if any, are likely to be subtle and clinically relevant only in the context of severe, pre-existing deficiency. The potential benefits should be weighed against the lack of a clear physiological function and the inconsistent evidence base [51,52].

Cr influences insulin signaling primarily by promoting the activation of the insulin receptor and IRS-1; enhancing the PI3K/Akt pathway, increasing GLUT4 translocation; modulating PPARγ, thereby improving gene expression related to insulin sensitivity, acting as a molecular cofactor in glucose tolerance, and reducing oxidative stress, thereby protecting the signaling cascade [51,52].

**Figure 2:**
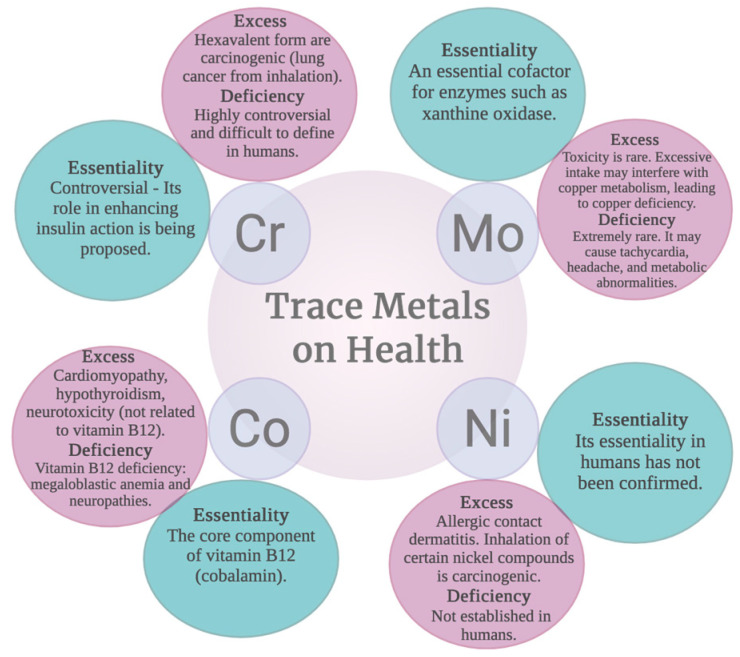
Summary of the essentiality, excess, and deficiency of the trace metals Cr, Mo, Ni, and Co. Created in https://BioRender.com.

Cr deficiency, especially in T2DM with cardiovascular complications, is associated with reduced GLUT4 expression and functional impairment of these mechanisms, which contributes to insulin resistance. However, further research is needed to confirm the causal role of these effects [51,52]. Figure 2 summarizes the toxic and essential effects of Cr.

#### 2.5.5. Chromium Intoxication

Acute exposures to Cr(VI) by inhalation or orally manifest as respiratory distress, vomiting, diarrhea, hemorrhage, dizziness, and there can even be multiorgan failure, coma, and death; dermal exposure can generate ulcers and burns. If exposure is chronic, asthma can develop, lung conditions including lung cancer, allergic dermatitis, kidney and liver failure, anemia, hemolysis, and gastric cancer [53]. Figure 2 summarizes the toxic and essential effects of Cr.

### 2.6. Molybdenum

Mo, named from the Greek molybdos (“similar to lead”), was often confused with lead and graphite [54].

#### 2.6.1. Chemical Properties and Metabolism

Biologically, Mo’s catalytic role in nitrogen fixation was recognized in the 1930s, and its essential function in molybdenum cofactor (Moco)–dependent enzymes, present since the last universal common ancestor (LUCA), was later established [55].

In nature, it occurs mainly in minerals and aquatic systems as the bioavailable molybdate anion (MoO_4_^2−^), exhibiting oxidation states from −4 to +6, with Mo(IV) and Mo(VI) predominating [56,57]. In aqueous environments, molybdate is soluble and can polymerize into polymolybdate species, as well as form thiomolybdates upon reaction with hydrogen sulfide [58]. It can also coordinate with oxygen and sulfur donors and adsorb onto manganese oxides and iron oxyhydroxides, depending on pH [59].

Mo is efficiently absorbed in the gastrointestinal tract (50–93%) as MoO_4_^2−^, distributed primarily in the liver and kidneys, and excreted via urine [56]. Its interaction with copper (Cu) can induce secondary deficiency (molybdenosis), and molybdoenzymes can generate reactive oxygen species (ROS), such as superoxide anion (O_2_^−^) and hydrogen peroxide (H_2_O_2_), which participate in cellular signaling or may induce oxidative stress [60].

#### 2.6.2. Sources of Molybdenum

Mo originates from both natural and anthropogenic sources, with the latter increasingly recognized as an environmental contaminant. Non-mineral Mo concentrations in the Earth’s surface average 1–3 ppm [61], and in the lithosphere, Mo can accumulate in porphyry ore deposits, igneous bodies, or residual melts. The most commercially important minerals are molybdenite, ferrimolybdenite, and jordisite, while minor minerals such as powellite, wulfenite, and ilsemanite (a molybdenum oxysulfate) have limited economic relevance [62]. In aquatic systems, Mo is released primarily through oxidative weathering and hydrolysis of primary minerals, making it the most abundant transition metal in seawater, typically at concentrations below 10 μg/L [63]. In rivers and lakes, total Mo levels generally remain below 2–3 μg/L [62].

For humans, dietary intake represents the main source of Mo, with an estimated daily consumption of 0.1–0.5 mg. Foods rich in Mo include legumes (particularly beans, peas, and lentils), cereal grains, liver, kidneys, milk, and certain leafy vegetables [64]. The reported toxic effects of Mo in humans are variable; however, exceeding the upper dietary limit of 2 mg per day may lead to long-term toxic effects. Mo human toxicity concentrations. Major anthropogenic sources of Mo in the environment include coal combustion, municipal sewage sludge, and industrial or mining operations. The increased use of Mo-containing products in industrial applications has significantly elevated the risk of exposure [65]. The mining of tungsten is considered the primary source of Mo contamination, as its waste can simultaneously release Mo and cadmium (Cd) into the environment. Reported concentrations range from 260–390 mg/kg in soils, up to 3800 μg/L in rivers, 0.4 mg/L in surface waters, and up to 25 mg/L in groundwater [66].

In addition to coal combustion residues, other industrial sources include the manufacture of fertilizers and the use of Mo in steel alloys, pigments, lubricants, and chemical catalysts [67].

#### 2.6.3. Biological Functions of Molybdenum

The essential biological function of molybdenum is attributed to its incorporation into the molybdenum cofactor (Moco), a pterin-based structure (molybdopterin, MPT) that coordinates the Mo ion [3]. Molybdoenzymes are key catalysts in the carbon, nitrogen, and sulfur cycles, performing redox reactions coupled with oxygen transfer, with Mo alternating between the IV and VI oxidation states [55]. In humans and animals, four main molybdoenzymes have been identified [54]:

- Sulfite Oxidase (SO): Metabolizes sulfur-containing amino acids (such as cysteine and methionine) by oxidizing toxic sulfite into sulfate. It is essential for human life; its deficiency causes neurological damage, seizures, and neonatal death.
- Xanthine Oxidoreductase (XOR): Metabolizes purines. Converts hypoxanthine to xanthine and xanthine to uric acid. It is important for homeostatic control, although its deficiency can cause kidney stones due to xanthine accumulation, and its excess contributes to gout and inflammation.
- Aldehyde Oxidase (AOX): Metabolizes aldehydes and exogenous compounds (drugs, toxins). Its precise endogenous substrates are still a matter of speculation, but it participates in the detoxification of xenobiotics.
- Mitochondrial Amidoxime Reductase 1 and 2 (mARC1/2). Reduces N-hydroxylated compounds (such as prodrugs and mutagens). It also participates in the metabolism of nitrites to nitric oxide. It has been implicated in lipogenesis and fatty liver diseases.

In higher plants such as *Arabidopsis thaliana*, molybdenum homeostasis depends on a delicate network of specialized transporters, with the MOT1 family playing a crucial role in root uptake and vacuolar mobilization. While MOT1.1 acts as a high-affinity importer in the root plasma membrane, MOT1.2, located in the tonoplast, mobilizes molybdenum stored in the vacuole to supply the biosynthesis of the molybdenum cofactor (Moco). However, knowledge regarding the export and detoxification mechanisms of this metal remains limited. In this context, the recent discovery in the microalga Chlamydomonas reinhardtii of the first ABC transporter involved in the export and detoxification of molybdate is particularly relevant [68]. This finding fills an important gap, as in plants such as Arabidopsis, a molybdenum exporter at the plasma membrane level has not yet been identified, and the mechanisms for preventing toxicity from excess metal are poorly understood. The existence of this ABC transporter in *C. reinhardtii* suggests that, in photosynthetic organisms, there may be a conserved detoxification pathway that complements glutathione-mediated vacuolar storage (proposed for plants). However, it is critical to note that this export function has not been confirmed in angiosperms, where the primary strategy appears to be vacuolar sequestration via GSH-molybdate complexes, rather than active export out of the cell. Therefore, future studies should explore whether homologs of this ABC transporter in terrestrial plants are involved in tolerance to high molybdenum concentrations, or whether, conversely, plants have opted for alternative intracellular compartmentalization pathways to manage excess amounts of this essential but potentially toxic trace element [68,69].

#### 2.6.4. Molybdenum Deficiency

Mo deficiency in humans is an infrequent phenomenon (because Mo is present in a wide variety of foods; therefore, acquired nutritional deficiency is nonexistent). However, the most relevant and documented form is MoCD (molybdenum cofactor deficiency), which is an autosomal recessive genetic disorder that prevents the synthesis of this factor, which is essential for the function of many enzymes [70,71]. The incidence of this disorder is very low; it is estimated that 1 in 200,000 live newborns are affected [72]. This deficiency is caused by sulfite accumulation and generates progressive neurological damage, as described below (Figure 2).

#### 2.6.5. Molybdenum Intoxication

Deficiency-induced damage (MoCD/sulfite oxidase deficiency). In MoCD, the loss of sulfite oxidase (SOX) activity leads to the accumulation of sulfite and S-sulfocysteine (SSC), highly reactive compounds that damage DNA and protein disulfide bonds [73]. These effects explain the severe neurodegeneration and lens dislocation observed in affected individuals [74]. The lack of inorganic sulfate also limits the synthesis of sulfated compounds essential for brain development.

Together, these mechanisms result in a neonatal presentation characterized by seizures and rapid neurological deterioration [75].

Excess-induced damage (molybdenosis) and copper antagonism. In ruminants, molybdenosis leads to secondary copper (Cu) deficiency [57]. Molybdate reacts with sulfide in the rumen to form thiomolybdates that bind Cu, reducing its absorption and increasing biliary excretion. This decreases ceruloplasmin and superoxide dismutase (SOD) activity, impairing iron transport and antioxidant defenses, and promoting anemia and oxidative tissue damage [3].

Oxidative stress and cellular damage. In monogastric species and cellular models, Mo excess alone or in combination with cadmium (Cd) has been reported to induce oxidative stress in the liver, kidney, lung, and testes [65]. This condition is characterized by increased lipid peroxidation (MDA) and reduced antioxidant enzyme activities (SOD, CAT, GPx), accompanied by mitochondrial dysfunction and apoptosis [65,67]. Co-exposure to Cd activates regulated cell death pathways such as ferroptosis, necroptosis, and pyroptosis, mediated by the NLRP3/Caspase-1 axis [76]. Co-exposure to molybdenum and cadmium in the testis induces oxidative stress due to an imbalance in trace elements (decreases in Cu, Zn, Fe, Se) and a decline in antioxidant capacity, which triggers DNA damage (8-OHdG, γ-H2AX) and activation of the ATM/AMPK/mTOR pathway, with consequent mTOR inhibition and increased autophagy (LC3-II, Beclin-1, Atg5) as a protective mechanism. Concurrently, in other organs, the NLRP3/Caspase-1 inflammatory pathway is activated, leading to pyroptosis, while genotoxic damage also causes cell cycle arrest at the G1/S phase. Collectively, oxidative stress acts as a central axis linking autophagy, inflammation, and cell cycle disruption in response to the combined toxicity of both metals [76]. Figure 2 summarizes the toxic and essential effects of Mo.

### 2.7. Cobalt

German miners called this mineral *kobold* because, when they tried to smelt it, it released toxic fumes (arsenic) and they did not obtain the expected copper, contaminating other metals. Its biological importance is revealed because it is the central metal of vitamin B12. Today it is used in alloys and medical devices, although its toxicity persists, especially in metal-on-metal prostheses [77].

#### 2.7.1. Chemical Properties and Metabolism

Co is essential as the redox-active core of vitamin B12, and it facilitates key reactions in methionine synthase and methylmalonyl-CoA mutase for DNA, in hematopoiesis, and in neurological function. However, Co^2+^ generates ROS, displaces cellular metals, and its toxicity depends on solubility, with CoCl_2_ being particularly cytotoxic [78]. Cobalt is absorbed in the jejunum, competing with Fe^2+^ (5–45%). It circulates mostly bound to albumin, and the free fraction is the toxic one. It is distributed to the liver, kidney, heart, and spleen, and it is excreted via the renal route. Its incorporation into cobalamin depends on chelatases and on chaperones such as CobW [79].

#### 2.7.2. Sources of Cobalt

Co comes from natural, dietary, occupational, and medical sources. Naturally, it is found in soils, rocks, and water at low concentrations, although mining areas can present considerably elevated levels. Diet is the main source for the general population, especially through foods rich in vitamin B12 (meats, dairy products, and fish), as well as chocolate, coffee, nuts, and green leafy vegetables. In the occupational setting, cobalt is used in the manufacture of hard metals and superalloys, pigments, cements, plastics, and batteries, as well as in the recycling of electronic waste. Inhalation of fine particles or dermal contact are frequent exposure routes [79]. In consumer products, it can be present in inexpensive jewelry, tanned leather, cosmetics, and certain pigments used in tattoos, constituting a common cause of allergic dermatitis. In medicine, the main risk comes from wear of metal-on-metal prostheses that release Co^2+^ ions into circulation, in addition to its historical use as an anti-anemic agent and its current use in radiotherapy [80].

#### 2.7.3. Biological Functions

The essential function of cobalt stems from its role as the central metal in vitamin B12, which is necessary for the activity of methionine synthase and methylmalonyl-CoA mutase. These enzymes are key to DNA synthesis, erythropoiesis, and the integrity of the nervous system. In prokaryotes, cobamides perform additional functions as metabolic cofactors, light sensors, and gene regulators. Furthermore, Co^2+^ ions can stabilize HIF-1α and promote angiogenesis, which has applications in biomaterials; however, this same reactivity explains their cytotoxic potential [81]. In the general population, total dietary intake of cobalt (including inorganic cobalt present in food and water) ranges from 5 to 50 µg/day, equivalent to 0.13–0.48 µg/kg/day. Established safety limits, such as the AFSSA’s acceptable daily intake, range from 1.6 to 8 µg/kg/day. Concentrations of 300–700 μg/L are associated with reversible hematological and endocrine effects, while levels ≥ 700–800 μg/L can cause more severe neurological, reproductive, and cardiac effects [82].

#### 2.7.4. Cobalt Deficiency

Cobalt deficiency occurs exclusively as vitamin B12 deficiency. Clinically, it causes megaloblastic anemia, macrocytosis, and ineffective erythropoiesis. At the neurological level, it leads to neuropathy due to cobalamin deficiency, cognitive deterioration, motor alterations, and myelin damage. Biochemically, it is characterized by hyperhomocysteinemia and methylmalonic aciduria, which increase the risk of cardiovascular diseases, neurodegenerative diseases, and bone disorders. People with vegetarian or vegan diets are especially vulnerable [83] (Figure 2).

#### 2.7.5. Cobalt Intoxication

Excess cobalt, especially in the form of free Co^2+^ ion, produces a multisystemic syndrome known as cobaltism. The most frequent manifestations are neurological: hearing and vision loss, tinnitus, vertigo, and peripheral neuropathy with paresthesias and weakness. At the cardiovascular level it can cause cardiomyopathy and tachycardia; in the endocrine system, hypothyroidism and goiter due to altered iodine uptake. It also induces polycythemia by mimicking hypoxia and stimulating erythropoietic activity. Exposure causes contact dermatitis, asthma, and pneumoconiosis. Some forms of cobalt are classified as probably carcinogenic due to oxidative stress and DNA damage [82]. Figure 2 summarizes the toxic and essential effects of Co.

### 2.8. Nickel

In 1751, the Swedish chemist Axel Cronstedt was the first to obtain it in its purified form, since previously it had been misidentified as a copper mineral called kupfernickel, or “the devil’s copper” [84].

#### 2.8.1. Chemical Properties and Metabolism

It occupies 24th place among the most abundant elements in the Earth’s crust [85], it has a silvery-white color, and it occurs in multiple oxidation states (−1 to +4), with the divalent state (+2) being the one mainly found in biological systems [84].

Ni can be absorbed by inhalation, oral, and dermal routes, it is transported through the bloodstream bound to albumin and to metalloproteins, it is widely distributed in the body in greater amounts in the lungs, thyroid gland, and adrenal glands, and in smaller amounts in the brain, kidneys, heart, liver, spleen, and pancreas, and it is excreted through urine and breast milk [86].

#### 2.8.2. Sources of Nickel

Ni is present in the environment in combination with oxygen and sulfur. Each year, 30,000 tons of this metal are released from natural sources such as volcanic emissions, marine aerosols, and soil erosion, among others. Anthropogenic sources such as mining, industry, and the burning of fossil fuels contribute almost twice as many tons emitted annually as natural sources [87]. Due to its physicochemical characteristics such as high ductility, shine, resistance to high temperatures, corrosion, and oxidation, this metal has a wide range of applications in modern metallurgy [88]. In foods, it is found in greater amounts in vegetables such as legumes and spinach, also in nuts, cocoa, and baking powder [89].

#### 2.8.3. Biological Functions of Nickel

In biological systems, a dual role of Ni has been evidenced: on the one hand, it is an essential micronutrient that catalyzes diverse metalloenzymes for microorganisms, plants, and animal species such as humans (since it is part of proteins and amino acids) [90].

#### 2.8.4. Nickel Deficiency

Ni deficiency is a rare condition, since it is absorbed through the diet; however, its depletion has been associated with perinatal mortality, low absorption of other trace elements such as Fe, Cu and Zn with modifications in cell morphology, lipid metabolism, and glucose metabolism [91] (Figure 2).

#### 2.8.5. Nickel Intoxication

In contrast, diverse studies have evidenced the toxic role of this metal, associated mainly with inhalation exposure to high concentrations (≥1 mg Ni/m^3^) of soluble compounds and to mixtures of nickel compounds (≥10 mg Ni/m^3^). Consequently, the International Agency for Research on Cancer (IARC) classified soluble and insoluble nickel compounds as Group 1 (carcinogenic to humans), and nickel alloys as Group 2B (possibly carcinogenic to humans) [92]. High and prolonged exposure to nickel compounds—resulting from their excessive release during fossil fuel combustion and manufacturing processes—generates a risk mainly for occupationally exposed individuals [8]. The documented health effects include [87]: Hypersensitivity and dermatological effects: severe skin allergies; respiratory and general symptoms: difficulty breathing, headache, and cough; gastrointestinal symptoms: nausea, vomiting, and diarrhea; systemic toxicity: hepatotoxic, nephrotoxic, hemotoxic, immunotoxic, reprotoxic, and pneumotoxic agent; genotoxic and carcinogenic potential: in addition to its carcinogenic potential, it has been identified as genotoxic; dietary exposure: a lifestyle that includes a diet with high Ni content also results in serious and chronic health problems.

Figure 1 summarizes the toxic and essential effects of Ni.

## 3. Non-Essential Metals

Unlike the metals described above, this category includes metals that, to date, have no known essential role in human metabolism, growth, or reproduction (although they may play such a role in some other organisms). In general, their presence in biological systems, even at very low concentrations, is often associated with toxic effects (such as enzyme inhibition, genetic damage, and other dysfunctions), although in certain cases the dose–response relationship may be more complex, and thresholds or exceptions may exist depending on the biological context and the chemical form of the element.

In this section, we will explore the effects of three metals present in our environment: lead (Pb), cadmium (Cd), and vanadium (V), which are generated inevitably by human activities such as mining, the metallurgical industry, and the burning of fossil fuels, among others.

In this way, the presence of these metallic elements is linked to crucial anthropogenic activities, and, at the same time, they represent a threat to health.

### 3.1. Lead

Pb, valued and used since antiquity for its unique properties, continues to be a silent problem for humanity. Some of its characteristics, such as ductility, malleability, low hardness, and resistance to atmospheric corrosion, facilitated its use in cosmetics, utensils, ornaments, writing, coin minting, and construction materials [93]. Historically, its versatility in different applications translated into widespread and chronic exposure, although its toxicity has been fully recognized only in recent times. The greatest source of anthropogenic dispersion of the metal occurred during the 20th century with the extensive use of lead in gasoline, which significantly increased global environmental contamination, mainly in urban areas. Although many of its applications have been restricted by health regulations, the persistence of Pb in the environment keeps it a major public health challenge [93,94].

#### 3.1.1. Chemical Properties and Metabolism

Lead is a toxic dark gray heavy metal, inert, and with the capacity to attenuate ionizing radiation; chemically, its toxicity lies in its ability for molecular mimicry of essential divalent cations such as Ca, Mg, Zn, and Fe, which it displaces from their active sites in biomolecules. Pb binds to proteins with high affinity, particularly to sulfhydryl groups (−SH) [94].

While it is true that lead toxicity is dose-dependent, no safe threshold has been identified below which there are no adverse effects, particularly neurocognitive damage in children. Therefore, the WHO guideline establishes a pragmatic threshold for clinical intervention (identifying and eliminating the source) when blood lead levels are ≥5 µg/dL. This is a strong recommendation based on high-certainty evidence regarding toxicity at low levels, emphasizing that primary prevention is the most important strategy [95].

Pb has different routes of entry; the most efficient is inhalation, with absorption > 90% for fine particles, while ingestion varies between 10% and 50%, and the least efficient is dermal absorption, at approximately <1% [94,95].

Lead’s high affinity for red blood cells has fundamental implications: first, regarding its transport and half-life, since lead bound to red blood cells circulates in the blood for ~40 days, which prevents its rapid elimination and allows it to be distributed to soft tissues (liver, kidneys, brain) and subsequently to bone; it is subsequently stored in bone because lead is deposited in bone tissue by displacing calcium; it remains there for decades, with a half-life of up to 30 years, causing the bone to act as an internal reservoir that slowly releases lead. Furthermore, conditions such as pregnancy, breastfeeding, or osteoporosis can reactivate bone release, causing blood levels to rise again.

Since lead inhibits enzymes such as ALAD and ferrochelatase within erythrocytes, hemoglobin synthesis is blocked, leading to microcytic anemia. The binding of lead to erythrocytes explains its long persistence in the body, its chronic toxicity (especially anemia), and the need for diagnostic methods beyond blood lead levels to assess total body burden. Excretion, mainly renal, is slow, favoring chronic accumulation [94,96].

#### 3.1.2. Sources of Exposure to Lead

Pb sources can be classified as natural, which include geological processes such as erosion, leaching, weathering, and volcanic activity. However, these contributions are minimal compared with those of anthropogenic origin [97,98]. Among anthropogenic sources are industrial and occupational sources, such as mining, smelting, the manufacture and recycling of batteries, pigments, solders, and munitions; as well as domestic sources, which include lead-based paints, pipes and solders in drinking-water systems, and glazed ceramics [98]. Despite regulations, the non-biodegradable nature of lead means that historical sources continue to be persistent contaminants [97,98], so lead remains a cause for environmental concern, as no safe concentration for human health has been established, and children are more susceptible to lead absorption and poisoning [99].

#### 3.1.3. Lead Intoxication

Pb intoxication (saturnism or plumbism) manifests through a broad spectrum of damage that results from the substitution of Pb for essential cations. Toxicity occurs at blood concentrations equal to or greater than 5 µg/dL, although this level is only a reference point for health intervention, not a safety limit [95,100].

Damage mechanism: Pb interferes with multiple biochemical pathways, altering normal functions of the hematopoietic system by inhibiting some enzymes that participate in heme-group synthesis, such as δ-aminolevulinate dehydratase (δ-ALAD), causing anemia and altering erythropoiesis. It also produces oxidative stress by inducing an increase in reactive oxygen species (ROS), causing direct damage to lipids, proteins, and DNA, and simultaneously depleting cellular antioxidants such as glutathione (GSH) [96]. In addition, it produces neurotoxicity by mimicking Ca, interfering with neurotransmitter release and cellular signaling.

Chronic damage in the central nervous system, even from low exposures, translates into cognitive alterations, reduction in IQ, and neuropathies [95,100].

Manifestations from chronic or acute intoxication are multisystemic: according to the WHO guidelines for the clinical management of lead exposure, the manifestations of acute or chronic lead poisoning are multisystemic and, at the neurological level, include cognitive impairments (such as deficits in IQ, attention, memory, and reasoning), severe encephalopathy that can progress to seizures, coma, and even death, as well as peripheral neuropathy, which manifests as decreased nerve conduction velocity and, in severe cases, radial palsy (drooping of the wrist or foot). These effects are particularly significant in children, in whom exposure to low levels of lead is associated with irreversible long-term neurobehavioral damage [95]. Cardiovascular alterations can also be observed, such as hypertension, where it has been estimated that Pb exposure has caused millions of premature deaths in adults from cardiovascular disease. Renal alterations such as chronic nephropathy and reproductive alterations such as infertility and adverse effects on fetal development are also observed [96,100].

**Figure 3:**
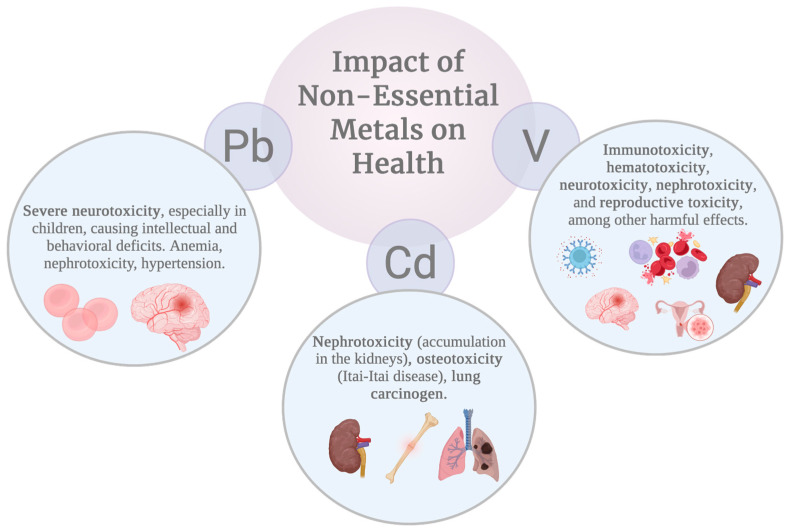
Summary of the health effects of the non-essential metals Pb, Cd, and V. Created in https://BioRender.com.

Diagnosis is based on measuring Pb in blood. Treatment consists of eliminating the exposure source and, in moderate or severe cases, administering chelation therapy with succimer, calcium disodium edetate, or dimercaprol [95,101] (Figure 3).

### 3.2. Cadmium

Cd is a metal that was first identified by Friedrich Stromeyer in 1817, as an impurity in zinc carbonate [102]. Naturally, this metal is found in soils, in Zn, Pb and Cu minerals, and in water. Its concentration in the Earth’s crust ranges from 0.1 to 0.2 ppm, although in phosphorites and marine phosphates it can reach 500 ppm [103].

#### 3.2.1. Chemical Properties and Metabolism

This toxic element is a transition metal in the form of a silvery-white or slightly bluish powder that has also been classified as a heavy metal. It belongs to group IIB of the periodic table, with atomic number 48, odorless, malleable, flammable, and insoluble in water [104]. It has relatively low melting and boiling points, which is convenient for using it in different industrial applications, such as the manufacture of pigments and batteries, as an anticorrosive agent, a stabilizer in PVC products, in alloys with other metals, in infrared detection, as well as in the electroplating industry [102].

#### 3.2.2. Sources of Exposure to Cadmium

Weathering of Cd-rich rocks and volcanic eruptions are the main natural sources of emission of this metal into the atmosphere. However, it is human activities such as the burning of fossil fuels, leaching in landfills, waste from mining activity, waste from agricultural activity due to the use of phosphate fertilizers with Cd traces, as well as the inadequate disposal of electronic waste, that emit the greatest amount of this metal [105].

Once released into the atmosphere, whether as an aerosol, gas, or suspended particles, Cd is distributed in air, water, and soil, and it enters the organism mainly through food contamination. According to various international agencies, permissible limits of Cd ingestion have been established; for example, the FAO (Food and Agriculture Organization) together with the WHO recommend a permissible limit of 25 µg Cd/kg body weight/month [104]. On the other hand, exposure limits through inhalation in workplace environments have been established. According to the International Cadmium Association (ICdA), the OEL (Occupational Exposure Limit) should not exceed 4 µg/m^3^ of Cd by inhalation exposure [105].

#### 3.2.3. Biological Functions

Cd has no known biological activities in organisms. However, it has been documented that it induces numerous toxic effects due to its ability to mimic essential divalent metals, such as Mg, Zn, Ca, Fe, and Cu, because its predominant oxidation state is +2. On the other hand, it binds to sulfhydryl groups of proteins, which alters their function and can result in alterations in their folding and in metabolic disorders [104]. It is important to consider that, even at low concentrations in the organism, Cd is capable of triggering significant damage because it is not biotransformed into less toxic species and because the effectiveness of chelating agents is low, its excretion is inefficient, which promotes its bioaccumulation, mainly in the kidneys [104,106]. It has been documented that, in the human body, Cd half-life ranges between 16 and 30 years [107].

#### 3.2.4. Cadmium Deficiency

Cd is not an essential element for the body, so there are no diseases associated with a deficiency of this metal. Medical and nutritional concerns focus solely on the risks associated with its toxicity and bioaccumulation.

#### 3.2.5. Cadmium Intoxication

Evidence has shown that exposure to Cd produces damage to different organs, which is related to the development of respiratory, neurological, and cardiovascular diseases, kidney and bone damage, teratogenic effects, and various types of cancer, among others [106]. The first evidence of the damage potential from Cd exposure dates to 1858, when it was identified that those exposed to polishing agents developed respiratory and gastrointestinal problems. On the other hand, the first toxicological studies with Cd were carried out in 1919, and since then, they have continued to be documented [108] (Figure 3).

A summary of some of the most important damages that have been documented in different systems of the human body produced by exposure to Cd is included below:

- Renal system: Nephrotoxicity is the most relevant type of damage triggered by exposure to Cd, mainly because this metal accumulates especially in the renal cortex due to its interaction with metallothioneins and its ability to mimic divalent ions. It has been reported that chronic exposure to this metal triggers renal tubular damage with proteinuria as the first sign, followed by glucosuria, aminoaciduria, increased excretion of Ca and phosphorus (P), and finally chronic kidney disease [102,103].
- Respiratory system: It is related to the appearance of cough, wheezing, lung inflammation associated with the development of chronic obstructive pulmonary disease and cancer [109], anosmia, chronic rhinitis, destruction of the olfactory epithelium, respiratory stress, emphysema, bronchitis, and alteration of lung function [104].
- Cardiovascular system: It has been related to the development of hypertension, endothelial dysfunction, and atherosclerosis, which increases the risk of other cardiovascular conditions [109].
- Skeletal system: Cd is related to a reduction in bone density and mineralization because it interferes with Ca metabolism, and it can also produce intense bone pain, osteomalacia, osteoporosis, as well as the development of Itai-itai disease, which occurs as a consequence of severe Cd intoxication and presents with intense pain, osteoporosis, osteomalacia, and severe renal dysfunction [103,107].
- Nervous system: Studies indicate that exposure to Cd produces aberrant behavior, as well as a decrease in IQ, in both children and adults, since it has been reported that this metal is able to cross the blood–brain barrier, in addition to accumulating in epithelial cells of the choroid plexuses [108].
- Reproductive systems: The effect of exposure to Cd has been described mainly in the testes, in which morphological changes have been found in spermatogenic cells, a decrease in testosterone synthesis, disrupts spermatogenesis, as well as an alteration in prostatic function [108]. In women, Cd alters ovarian hormone production and produces hemorrhagic changes in the ovaries [104].
- Teratogenesis: Cd can affect fetal development because it crosses the placental barrier and can also reach the fetus through breast milk [104].
- Cancer: The IARC classifies cadmium as a human carcinogen (Group 1) based on sufficient evidence, primarily from lung cancer among workers. In addition, the IARC noted positive associations with cancer risk in the prostate, kidney, bladder, breast, and endometrium [105].

### 3.3. Vanadium

Vanadium was discovered in 1801 by Andrés Manuel del Río in Mexico, who named it panchromium and later erythronium, but he was not given credit for the discovery. In 1830, Nils Gabriel Sefström rediscovered it and named it vanadium in honor of the goddess Vanadis. Although it was confirmed to be the same element, the name vanadium had already become established [110].

#### 3.3.1. Chemical Properties and Metabolism

Vanadium is a transition metal that exists in oxidation states ranging from −1 to +5. It is the 21st most abundant element in the Earth’s crust and the second most abundant in seawater [111]. It is a ubiquitous metal that is present in soil, petroleum, water, and air [112].

Vanadium can enter the organism orally (food and water) and by inhalation (because it is present as an atmospheric contaminant); oral absorption is poor, 1–2%, whereas when inhaled, absorption can reach up to 90% [113]. Once it enters the organism, the most abundant V species are vanadate and vanadyl, which bind to the plasma proteins transferrin and albumin. In the bloodstream it can reach all organs and tissues and accumulate preferentially, for example, in bone [111]. Vanadium is eliminated through urine (mainly) and through feces [113].

#### 3.3.2. Sources of Exposure to Vanadium

Vanadium is present in many foods; the foods with higher concentrations of this metal are black pepper, parsley, spinach, and mushrooms. Foods of marine origin contain more V than foods of terrestrial origin. A potentially important source of V is dietary supplements: doses have been reported ranging from 0.1 mg to more than 100 mg, and the latter greatly exceed levels considered safe. Vanadium is emitted into the atmosphere mainly by anthropogenic activities such as the burning of fossil fuels and the metallurgical industry. Natural sources (such as volcanic eruptions and marine aerosols) also contribute to environmental emissions [113].

#### 3.3.3. Biological Functions of Vanadium

V is an element that for decades has generated controversy because it raises a major contradiction, from toxicity to essentiality. The importance of this element as a micronutrient has still not been accepted unequivocally [114], and its essentiality in humans is still under discussion and needs to be elucidated [115]. It is believed that V is important for normal cellular function and development, since it is present in all tissues involved in glucose homeostasis, lipid metabolism, antioxidant functions, and as an immunomodulator; however, its role as a trace element has not been established in humans or in animals [116,117], but it is considered essential for various specific organisms [112].

Interesting examples of organisms that accumulate vanadium (although its presence does not seem to have any biological effect and no precise functions have been identified) are macroalgae, ascidians, some fan worms (because V is the second most abundant metal in the ocean), and mushrooms of the genus Amanita. It is considered that, since V is toxic, accumulation in these organisms could indicate that it is poisonous to their predators [118] and thus serve as a protective mechanism for them.

#### 3.3.4. Vanadium Deficiency

At present, the essentiality of this metal in humans is under discussion: it is believed that V is important for normal cellular function and development, since it is present in all tissues involved in glucose homeostasis, lipid metabolism, antioxidant functions, and as an immunomodulator; however, its role as an essential element has not been established in humans or in animals [116,117], but it is considered essential in most living beings [112].

On the other hand, V deficiency has been associated with physiological dysfunctions involved in thyroid, carbohydrate, and lipid metabolism, in addition to the fact that there are genes regulated by V, including: tumor necrosis factor-alpha (TNF-alpha), interleukin 8 (IL-8), activator protein-1 (AP-1), and mitogen-activated protein kinase (MAPK). This indicates that V could be close to being recognized as an essential element, in addition to the fact that it is also emerging as an anticancer agent [114].

#### 3.3.5. Vanadium Intoxication

V is associated with diverse pathogeneses: vanadium salts interfere with essential enzymes (ATPases, kinases, ribonucleases, and phosphatases) [114]. This interference is explained by the ability of vanadate to substitute for phosphate because it is a structural analog; therefore, vanadate can easily substitute for phosphate in enzymes such as phosphatases and kinases, and consequently, substitution of phosphate by vanadate leads to enzyme inhibition [118].

V is a metal that has been very interesting to researchers due to the duality of the effects it can cause in organisms. In this sense, the toxic potential of vanV when inhaled has been studied extensively by some research groups for several years [113,119], who, using an experimental murine model, have shown that this element generates adverse effects in all evaluated systems, since it causes immunotoxicity, hematotoxicity, neurotoxicity, nephrotoxicity, and reprotoxicity, among other harmful effects (Figure 3).

Furthermore, the ability of V to form organometallic compounds has contributed to research on its biological activity with a view to its applications in medicine as a potential metal-based drug [120], which has shown promising results in major diseases such as cancer, due to its wide range of mechanisms of action, including the induction of oxidative stress, DNA damage, cell cycle arrest, apoptosis induction, and regulation of the autophagy process. These compounds have been shown to be effective against the types of cancer with the highest incidence and mortality rates worldwide, such as lung and breast cancer [121].

## 4. Essential and Non-Essential Metals as Environmental Pollutants

As described above, many of the metals to which we are exposed are essential for life. However, their presence as environmental pollutants has become a cause for global concern. Metal pollution does not come only from natural sources such as volcanic activity and obvious anthropogenic sources such as the metallurgical industry, but also from common sources such as the use of public transportation. Other anthropogenic sources that are rarely considered and often underestimated are abandoned mines, which collectively contribute to increased concentrations to which organisms are exposed and which can be toxic to health.

The following table summarizes the main sources of contamination for the metals described above (Table 1).

## 5. Mechanisms of Action Common to Metals

Since redox-active transition metals (for example, Fe and Cu) can participate in electron-transfer reactions, their homeostasis must be carefully controlled. The catalytic behavior of redox metals that have escaped control, for example, through the Fenton reaction, gives rise to the formation of reactive hydroxyl radicals, which can cause damage to DNA, proteins, and membranes. Transition metals are an integral part of the active centers of numerous enzymes (for example, Cu, Zn-SOD, Mn-SOD, catalase) that catalyze chemical reactions at physiologically compatible rates. Both deficiency and excess of essential metals can lead to various diseases in an organism. Some typical complaints characterized by altered homeostasis of redox-active metals are neurological disorders (Alzheimer’s, Parkinson’s, and Huntington’s), mental health problems, cardiovascular diseases, cancer, and diabetes [3]. For example, iron overload has toxic effects mainly related to oxidative stress, since free iron can change oxidation state between Fe^2+^ and Fe^3+^ ions through the Fenton reaction, in which free radicals and reactive oxygen species are generated. This condition can damage lipids, proteins, and DNA, alter mitochondrial function, and even trigger different types of cell death, including ferroptosis [10,17]. In the liver, in addition to oxidative stress, an increase in TGF-β1 production has been demonstrated, which increases the risk of fibrosis [9]. Cr(VI) enters the cell actively using sulfate or phosphate transporters; intracellular reduction of Cr(VI) to Cr(III) by ascorbate and glutathione generates oxidative stress, resulting in the production of free radicals and reactive oxygen species, which alter signaling pathways, cause genotoxic damage with formation of adducts and micronuclei, and trigger cell death by apoptosis [122].

Some of the mechanisms of action by which excess Co causes damage are hypoxia mimicry: it stabilizes HIF-1α, induces erythropoietin, and promotes angiogenesis. Mismetallation: It displaces Fe, Zn, Mg, and Mn, altering Fe–S groups, inhibiting TCA cycle enzymes, and interfering with heme formation. Oxidative stress: It generates ROS that damage DNA and block its repair. Mitochondrial damage and neurotoxicity: It interferes with proteins that contain thiol groups and affects neurons of the retina and the cochlea. Local toxicity and carcinogenesis: Especially due to Co^2+^ released from metal-on-metal prostheses [80,83]. And by its deficiency it blocks essential enzymatic reactions by inactivating methionine synthase and methylmalonyl-CoA mutase. It causes accumulation of toxic metabolites, such as homocysteine and methylmalonate, with antiangiogenic and cytotoxic effects, and hematological and neurological damage is reflected in failure of DNA synthesis and neuronal deterioration [80].

**Figure 4:**
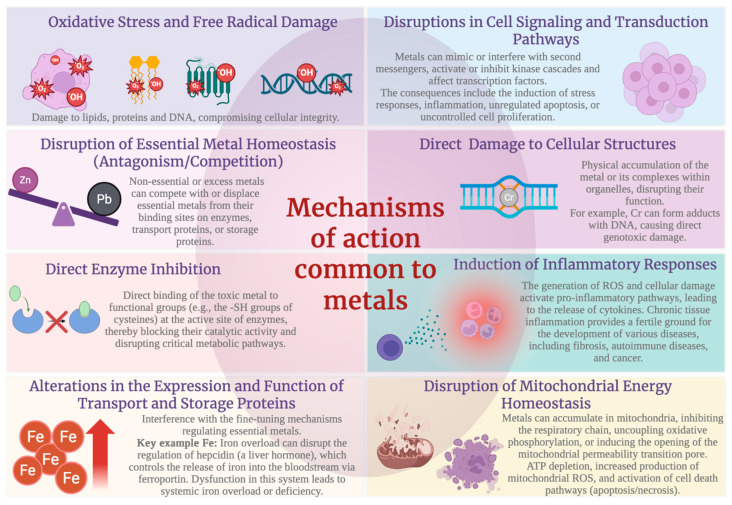
Summary of the main mechanisms of action common to metals. Created in https://BioRender.com.

In general, metals cause an imbalance between cellular oxidants and antioxidants, that is, they bring the cell into a state of oxidative stress; this is the most common and therefore the most documented mechanism. Another mechanism is interaction with protein sulfhydryl groups, because many metals have high affinity for thiol (–SH) groups found in the active sites of numerous enzymes and structural proteins, which can inactivate them. In addition, metals can cause DNA damage, that is, they are genotoxic, which can affect gene expression and increase the risk of cellular transformation and the generation of malignant neoplasms. Finally, metals can affect or alter cellular signaling pathways (Figure 4).

## 6. Conclusions

Metals in the organism have a paradoxical behavior that is summarized in the phrase coined by the famous physician and alchemist Paracelsus in the 16th century: All things are poison, and nothing exists without poison; only the dose makes a thing not a poison; in summary, the dose makes the poison (except Pb and Cd, which are always poisons). Deficiency of an essential metal causes a biological function to be affected. Homeostasis implies that this metal is present in precise and controlled amounts, which allows biological functions to be carried out normally. Excess occurs when the organism’s capacity to metabolize it, store it, or excrete it is exceeded, and therefore the metal becomes an agent that damages cells and tissues. In this regard, the main conclusions of the metals in this review are described below.

Iron: Fe is an essential metal, an indispensable component of many proteins, and it participates in diverse vital chemical reactions. Its deficiency is associated mainly with anemia; however, it can affect the functioning of other cells and increase the risk of neurological and cardiovascular disorders. On the other hand, iron overload also causes health problems, such as liver problems and an increased cardiovascular risk, among others.

Zinc: Both Zn deficiency and its excess are events that are rarely diagnosed, which does not exclude their existence. Given the trend toward consuming vegan and vegetarian diets, it would not be strange that deficiencies in Zn intake are more frequent than presumed. On the other hand, consumption of over-the-counter supplements must be considered in patients with symptoms suggestive of intoxication by this element.

Copper: It is an essential micronutrient whose biological value lies in its redox properties, Cu(I) and Cu(II), to serve as a cofactor for vital metalloenzymes. Its homeostasis is strictly controlled by the transporter proteins ATP7A and ATP7B. Dysregulation of this balance, either by deficiency or by overload, causes severe systemic and neurological pathologies, highlighting the need for precise molecular regulation to preserve human health.

Manganese: Mn is an essential metal for human survival, since it participates in numerous key processes in the organism. As it is present in foods, its deficiency is rare, but when it occurs, it can cause problems in growth, infertility, bone weakness, and metabolic alterations. The most concerning issue is excessive exposure to this element, since the main danger usually does not come from foods, but from exposure to dust or fumes that contain it. Mn accumulates mainly in the brain, especially in the basal ganglia, and causes oxidative stress, mitochondrial dysfunction, and neuroinflammation, which is reflected in a pathology known as manganism.

Chromium: Currently, Cr has been recognized as a trace element for humans due to its properties in glucose and lipid metabolism; however, its toxic potential that puts health at risk should not be overlooked.

Molybdenum: Although Mo is found naturally in the environment and in biological systems, modern industrial and mining activities have become important sources, since they release concentrated amounts of Mo that often exceed natural levels in soil, air, and water. These anthropogenic inputs re-enter biological systems through trophic chains, increasing the potential for bioaccumulation and toxic exposure. While Mo is essential for eukaryotic life, its toxicity depends largely on dose and can be aggravated by the presence of other heavy metals. Mo exerts a dual mechanism of toxicity: systemic interference with copper homeostasis, which alters sulfite oxidase (SOX) activity and impairs reproductive function, and direct cellular damage involving organelle dysfunction, oxidative stress, and pyroptosis mediated by activation of the NLRP3 inflammasome.

Cobalt: It represents a micronutrient with a profound biological duality: indispensable for life in its form integrated into vitamin B12, but highly toxic when it circulates as a free ion. Its redox chemistry and its ability to form metal–carbon bonds support essential metabolic functions, while at the same time explaining mechanisms of cellular toxicity. Its broad presence in industrial and medical applications requires rigorous control to balance its biological and therapeutic benefits with the prevention of toxicological risks.

Nickel: Ni can cause deterioration of human health in different organs through different mechanisms of action; among them, increased production of reactive nitrogen species has been identified, such as inducible nitric oxide synthase (iNOS) or nitric oxide (NO), triggering inflammatory processes and cell death by apoptosis. It also induces an increase in reactive oxygen species, and as one of the main mechanisms as a carcinogen, damage in DNA processing and repair has been identified through direct inhibition of enzymes and downregulation of expression of molecules involved in different repair systems. However, the work of different research groups continues to identify other mechanisms of action that cause the toxicity of this ubiquitous metal.

Lead: It is a persistent and toxic contaminant with no biological function. It accumulates in the body, causing irreversible damage in multiple organs. Because there is no safe level of exposure, it is vital to eliminate its sources to protect public health.

Cadmium: Cd is one of the most toxic metals for all living beings, and specifically in humans, even minimal concentrations produce important alterations in the organism, targeting vital organs such as the kidneys. Bioaccumulation of this metal makes treatment of these alterations difficult, and knowing its emission sources, as well as the damage it triggers, is important to reduce its harm.

Vanadium: V is an example of an element with a very fine boundary between an essential element, a therapeutic agent, and a dangerous contaminant. Its presence in the diet is in small amounts that are possibly harmless; environmental and occupational exposure, as well as the presence of high doses in dietary supplements, present important toxic risks to health.

In conclusion, we can say that humanity, with respect to metals, walks on a tightrope between deficiency that weakens and toxicity that poisons, with diet and the environment determining toward which side the balance tips between maintaining homeostasis and, therefore, health, or falling into disease. Understanding common mechanisms of damage is vital to developing therapeutic strategies, in addition to explaining the wide range of adverse health effects they generate. Furthermore, it is important to recall that these elements are part of the environmental pollution to which organisms are exposed daily, which is clearly harmful to health.

## Figures and Tables

**Table 1:** Main natural and anthropogenic sources of emissions of essential and non-essential metals into the environment.

Metal	Major Sources of Environmental and Human Pollution
Fe	Atmospheric iron in airborne particles generated by wear and tear on the tracks and wheels of the metro system; abandoned mines; and tool corrosion.
Zn	Industrial activities such as mining, metal smelting (steel), galvanizing, waste incineration, and the combustion of fossil fuels, sewage sludge, fertilizers, pesticides, and the leaching of construction materials.
Cu	Mining activities, smelters, manufacturing industries, the use of pesticides and fungicides, corrosion of household pipes, rock erosion, wildfires, and volcanic eruptions.
Mn	Fungicides (maneb and mancozeb), in fuel additives, and in the gasoline additive MMT (methylcyclopentadienyl manganese tricarbonyl), as well as being released into the atmosphere during mining activity and battery manufacturing.
Cr	Metallurgical and chemical industries; fumes from industries involved in electroplating and welding; dye manufacturing, leather processing, metal smelting, and the manufacture of catalysts for hydrocarbon processing; tobacco smoke.
Mo	Coal combustion, municipal sludge, industrial or mining activities, tungsten mining (which also releases Cd).
Co	Manufacture of hard metals and superalloys, pigments, cements, plastics, and batteries; recycling of electronic waste; inexpensive jewelry, tanned leather, cosmetics, and certain pigments used in tattoos. In medicine: wear of metal-on-metal prostheses that release Co^2+^ ions, in addition to its historical use as an anti-anemic agent and its current use in radiation therapy.
Ni	Mining, the metallurgical industry, and the burning of fossil fuels; volcanic emissions, marine aerosols, and soil erosion.
Pb	Erosion, leaching, weathering, and volcanic activity; industrial and occupational sources, such as mining, smelting, manufacturing, and the recycling of batteries, pigments, solder, and ammunition; as well as domestic sources, including lead-based paints, pipes and solder in drinking water systems, and glazed ceramics.
Cd	Weathering of cadmium-rich rocks and volcanic eruptions; burning of fossil fuels; leaching at landfills; waste from mining activities; agricultural waste resulting from the use of phosphate fertilizers containing traces of cadmium; improper disposal of electronic waste; and tobacco smoke.
V	Burning of fossil fuels and the metallurgical industry. Natural sources such as volcanic eruptions and marine aerosols.

## Data Availability

No new data were created or analyzed in this study. Data sharing is not applicable to this article.

## References

[1] Zhang Y., Ying H., Xu Y. (2019). Comparative genomics and metagenomics of the metallomes. Metallomics.

[2] Ramírez-Hernández J., Bonete M.J., Martínez-Espinosa R.M. (2015). Propuesta de una nueva clasificación de los oligoelementos para su aplicación en nutrición, oligoterapia, y otras estrategias terapéuticas. Nutr. Hosp..

[3] Jomova K., Makova M., Alomar S.Y., Alwasel S.H., Nepovimova E., Kuca K., Rhodes C.J., Valko M. (2022). Essential metals in health and disease. Chem. Biol. Interact..

[4] Guan D.-X., Yang C., Nriagu J.O. (2024). Editorial: The role of essential trace elements in health and disease. Front. Public Health.

[5] Rodríguez-Moro G., Ramírez-Acosta S., Arias-Borrego A., García-Barrera T., Gómez-Ariza J.L. (2018). Environmental Metallomics. Adv. Exp. Med. Biol..

[6] Zhu Y., Costa M. (2020). Metals and molecular carcinogenesis. Carcinogenesis.

[7] Sheftel A.D., Mason A.B., Ponka P. (2012). The long history of iron in the Universe and in health and disease. Biochim. Biophys. Acta.

[8] Liu H., Liu T., Chen S., Liu X., Li N., Huang T., Ma B., Liu X., Pan S., Zhang H. (2024). Biogeochemical cycles of iron: Processes, mechanisms, and environmental implications. Sci. Total Environ..

[9] Teschke R. (2024). Copper, Iron, Cadmium, and Arsenic, All Generated in the Universe: Elucidating Their Environmental Impact Risk on Human Health Including Clinical Liver Injury. Int. J. Mol. Sci..

[10] Gattermann N., Muckenthaler M.U., Kulozik A.E., Metzgeroth G., Hastka J. (2021). The Evaluation of Iron Deficiency and Iron Overload. Dtsch. Ärzteblatt Int..

[11] Ali M.K., Kim R.Y., Karim R., Mayall J.R., Martin K.L., Shahandeh A., Abbasian F., Starkey M.R., Loustaud-Ratti V., Johnstone D. (2017). Role of iron in the pathogenesis of respiratory disease. Int. J. Biochem. Cell Biol..

[12] Ji W., Zhao K., Liu C., Li X. (2022). Spatial characteristics of fine particulate matter in subway stations: Source apportionment and health risks. Environ. Pollut..

[13] Zhang M., Shao L., Jones T., Feng X., Ge S., Yang C., Cao Y., BéruBé K., Zhang D. (2022). Atmospheric iron particles in PM2.5 from a subway station, Beijing, China. Atmos. Environ..

[14] Zhou L., Zhang J., Zhu X., Xu D., Zheng S. (2024). The pollution characteristics and causes of dual sources–iron (Fe) in abandoned coal mines: A literature review. J. Clean. Prod..

[15] Szklarz M., Gontarz-Nowak K., Matuszewski W., Bandurska-Stankiewicz E. (2022). “Ferrocrinology”-Iron Is an Important Factor Involved in Gluco- and Lipocrinology. Nutrients.

[16] Słota M., Wąsik M., Stołtny T., Machoń-Grecka A., Kasperczyk S. (2022). Effects of environmental and occupational lead toxicity and its association with iron metabolism. Toxicol. Appl. Pharmacol..

[17] Nishikawa Y., Matsuo Y., Watanabe R., Miyazato M., Matsuo M., Nagahama Y., Tanaka H., Ooshio T., Goto M., Okada Y. (2023). Hepatocyte-specific damage in acute toxicity of sodium ferrous citrate: Presentation of a human autopsy case and experimental results in mice. Toxicol. Rep..

[18] Zhang Z., Weichenthal S., Kwong J.C., Burnett R.T., Hatzopoulou M., Jerrett M., Donkelaar A.V., Bai L., Martin R.V., Copes R. (2021). Long-term exposure to iron and copper in fine particulate air pollution and their combined impact on reactive oxygen species concentration in lung fluid: A population-based cohort study of cardiovascular disease incidence and mortality in Toronto, Canada. Int. J. Epidemiol..

[19] Kontoghiorghes G.J. (2023). Iron Load Toxicity in Medicine: From Molecular and Cellular Aspects to Clinical Implications. Int. J. Mol. Sci..

[20] International Zinc Association Historia del Zinc. https://latiza.zinc.org/zinc/zinc-historia/.

[21] Kambe T., Tsuji T., Hashimoto A., Itsumura N. (2015). The Physiological, Biochemical, and Molecular Roles of Zinc Transporters in Zinc Homeostasis and Metabolism. Physiol. Rev..

[22] Stiles L.I., Ferrao K., Mehta K.J. (2024). Role of zinc in health and disease. Clin. Exp. Med..

[23] Schoofs H., Schmit J., Rink L. (2024). Zinc Toxicity: Understanding the Limits. Molecules.

[24] National Library of Medicine Toxicological Profile of Zinc. https://www.ncbi.nlm.nih.gov/books/NBK600538/.

[25] Chen B., Yu P., Chan W.N., Xie F., Zhang Y., Liang L., Leung K.T., Lo K.W., Yu J., Tse G.M.K. (2024). Cellular zinc metabolism and zinc signaling: From biological functions to diseases and therapeutic targets. Signal Transduct. Target. Ther..

[26] National Institutes of Health Zinc. https://ods.od.nih.gov/factsheets/Zinc-HealthProfessional/#en2.

[27] Li J., Cao D., Huang Y., Chen B., Chen Z., Wang R., Dong Q., Wei Q., Liu L. (2022). Zinc Intakes and Health Outcomes: An Umbrella Review. Front. Nutr..

[28] Pearce M. (2019). The ‘Copper Age’—A History of the Concept. J. World Prehist..

[29] Uriu-Adams J.Y., Keen C.L. (2005). Copper, Oxidative Stress, and Human Health. Mol. Asp. Med..

[30] Jansen van Ryssen J.B., Bath G.F. (2025). Possible Factors Affecting the Bioavailability of Copper and the Copper Requirements of Wild, Free-Ranging African Herbivores: A Review. S. Afr. J. Anim. Sci..

[31] Liu Y., Zhu J., Xu L., Wang B., Lin W., Luo Y. (2022). Copper Regulation of Immune Response and Potential Implications for Treating Orthopedic Disorders. Front. Mol. Biosci..

[32] Harris E.D. (2001). Copper Homeostasis: The Role of Cellular Transporters. Nutr. Rev..

[33] Pierson H., Muchenditsi A., Kim B.E., Ralle M., Zachos N., Huster D., Lutsenko S. (2018). The Function of ATPase Copper Transporter ATP7B in Intestine. Gastroenterology.

[34] National Library of Medicine Toxicological Profile of Copper. https://www.ncbi.nlm.nih.gov/books/NBK610357/.

[35] Gambling L., McArdle H.J. (2004). Iron, Copper and Fetal Development. Proc. Nutr. Soc..

[36] Myint Z.W., Oo T.H., Thein K.Z., Tun A.M., Saeed H. (2018). Copper deficiency anemia: Review article. Ann. Hematol..

[37] Lutsenko S., Roy S., Tsvetkov P. (2025). Mammalian copper homeostasis: Physiological roles and molecular mechanisms. Physiol. Rev..

[38] Blanc P.D. (2018). The early history of manganese and the recognition of its neurotoxicity, 1837–1936. Neurotoxicology.

[39] Horning K.J., Caito S.W., Tipps K.G., Bowman A.B., Aschner M. (2015). Manganese Is Essential for Neuronal Health. Annu. Rev. Nutr..

[40] Zou J., Yerramilli R., Aydemir T.B. (2025). Gut to brain: Essential micronutrient and trace element manganese transport, function and toxicity. Front. Physiol..

[41] Magro G., Laterza V., Tosto F., Torrente A. (2025). Manganese Neurotoxicity: A Comprehensive Review of Pathophysiology and Inherited and Acquired Disorders. J. Xenobiotics.

[42] Gurol K.C., Jursa T., Cho E.J., Fast W., Dalby K.N., Smith D.R., Mukhopadhyay S. (2024). PHD2 enzyme is an intracellular manganese sensor that initiates the homeostatic response against elevated manganese. Proc. Natl. Acad. Sci. USA.

[43] Baj J., Flieger W., Barbachowska A., Kowalska B., Flieger M., Forma A., Teresiński G., Portincasa P., Buszewicz G., Radzikowska-Büchner E. (2023). Consequences of Disturbing Manganese Homeostasis. Int. J. Mol. Sci..

[44] Aschner M., Erikson K. (2017). Manganese. Adv. Nutr..

[45] Erikson K.M., Aschner M. (2019). Manganese: Its Role in Disease and Health. Met. Ions Life Sci..

[46] Lindner S., Lucchini R., Broberg K. (2022). Genetics and Epigenetics of Manganese Toxicity. Curr. Environ. Health Rep..

[47] Gonzalez-Villalva A., Rojas-Lemus M., López-Valdez N., Cervantes-Valencia M.E., Guerrero-Palomo G., Casarrubias-Tabarez B., Bizarro-Nevares P., Morales-Ricardes G., García-Peláez I., Ustarroz-Cano M. (2026). Metal Pollution in the Air and Its Effects on Vulnerable Populations: A Narrative Review. Int. J. Mol. Sci..

[48] Genchi G., Lauria G., Catalano A., Carocci A., Sinicropi M.S. (2021). The double face of metals: The intriguing case of chromium. Appl. Sci..

[49] Lunk H.J. (2015). Discovery, properties and applications of chromium and its compounds. ChemTexts.

[50] Vincent J.B., Lukaski H.C. (2018). Chromium. Adv. Nutr..

[51] Carvalho D.C., Coelho L.M., Acevedo M.S.M.S.F., Coelho N.M.M., De la Guardia M., Garrigues S. (2015). The oligoelements. Handbook of Mineral Elements in Food.

[52] Nayyar H., Bhatti A., John P. (2025). Impact of zinc and chromium deficiency on gene expression in type 2 diabetes mellitus. Sci. Rep..

[53] Balali-Mood M., Eizadi-Mood N., Hassanian-Moghaddam H., Etemad L., Moshiri M., Vahabzadeh M., Sadeghi M. (2025). Recent advances in the clinical management of intoxication by five heavy metals: Mercury, lead, chromium, cadmium and arsenic. Heliyon.

[54] Foteva V., Fisher J.J., Qiao Y., Smith R. (2023). Does the Micronutrient Molybdenum Have a Role in Gestational Complications and Placental Health?. Nutrients.

[55] Mayr S.J., Mendel R.R., Schwarz G. (2021). Molybdenum cofactor biology, evolution and deficiency. Biochim. Biophys. Acta Mol. Cell Res..

[56] Barceloux D.G. (1999). Molybdenum. J. Toxicol. Clin. Toxicol..

[57] Abejón R. (2022). An Overview to Technical Solutions for Molybdenum Removal: Perspective from the Analysis of the Scientific Literature on Molybdenum and Drinking Water (1990–2019). Water.

[58] Huang X.-Y., Hu D.-W., Zhao F.-J. (2022). Molybdenum: More than an essential element. J. Exp. Bot..

[59] Montero-Serrano J.C., Martínez-Santana M., Tribovillard N., Riboulleau A., Garbán G. (2009). Comportamiento geoquímico del molibdeno y sus isótopos en el ambiente sedimentario: Un resumen bibliográfico. Rev. Biol. Mar. Oceanogr..

[60] Mayers J., Hofman B., Sobiech I., Kwesiga M.P. (2024). Insights into the biocompatibility of biodegradable metallic molybdenum for cardiovascular applications-a critical review. Front. Bioeng. Biotechnol..

[61] Tambat V.S., Tseng Y.-S., Kumar P., Chen C.-W., Singhania R.R., Chang J.-S., Dong C.-D., Patel A.K. (2023). Effective and sustainable bioremediation of molybdenum pollutants from wastewaters by potential microalgae. Environ. Technol. Innov..

[62] Hlohowskyj S.R., Chappaz A., Dickson A.J. (2021). Molybdenum as a Paleoredox Proxy: Past, Present, and Future.

[63] Siebert C., Scholz F., Kuhnt W. (2021). A new view on the evolution of seawater molybdenum inventories before and during the Cretaceous Oceanic Anoxic Event 2. Chem. Geol..

[64] Albin M., Oskarsson A., Gunna F.N., Costa M. (2022). Chapter 23-Molybdenum. Handbook on the Toxicology of Metals.

[65] Cao P., Nie G., Luo J., Hu R., Li G., Hu G., Zhang C. (2022). Cadmium and molybdenum co-induce pyroptosis and apoptosis via the PTEN/PI3K/AKT axis in the livers of Shaoxing ducks (*Anas platyrhynchos*). Food Funct..

[66] Zhang C., Hu Z., Hu R., Pi S., Wei Z., Wang C., Yang F., Xing C., Nie G., Hu G. (2021). New insights into crosstalk between pyroptosis and autophagy co-induced by molybdenum and cadmium in duck renal tubular epithelial cells. J. Hazard. Mater..

[67] Cui T., Jiang W., Yang F., Luo J., Hu R., Cao H., Hu G., Zhang C. (2021). Molybdenum and cadmium co-induce hypothalamus toxicity in ducks via disturbing Nrf2-mediated defense response and triggering mitophagy. Ecotoxicol. Environ. Saf..

[68] Weber J.-N., Minner-Meinen R., Kaufholdt D. (2024). The Mechanisms of Molybdate Distribution and Homeostasis with Special Focus on the Model Plant *Arabidopsis thaliana*. Molecules.

[69] Weber J.N., Minner-Meinen R., Behnecke M., Biedendieck R., Hänsch V.G., Hercher T.W., Hertweck C., van den Hout L., Knüppel L., Sivov S. (2023). Moonlighting Arabidopsis molybdate transporter 2 family and GSH-complex formation facilitate molybdenum homeostasis. Commun. Biol..

[70] Adamus J.P., Ruszcynska A., Wyczalkowska-Tomasik A. (2024). Molybdenum’s role as an essential element in enzymes catabolizing redox reactions: A review. Biomolecules.

[71] Schwahn B.C., van Spronsen F., Misko A., Pavaine J., Holmes V., Spiegel R., Schwarz G., Wong F., Horman A., Pitt J. (2024). Consensus guidelines for the diagnosis and management of isolated sulfite oxidase deficiency and molybdenum cofactor deficiencies. J. Inherit. Metab. Dis..

[72] Sadek A.A., Aladawy M.A., Magdy R.M., Fouad M.F., Mansour T.M.M., Gad E.F., Abdelkreem E. (2025). Urinary stones as the initial presentation in a child with late-onset molybdenum cofactor deficiency type B: A case report and review of literature. Egypt. J. Med. Hum. Genet..

[73] Johannes L., Fu C.-Y., Schwarz G. (2022). Molybdenum Cofactor Deficiency in Humans. Molecules.

[74] Anke M., Seifert M. (2010). The biological and toxicological importance of molybdenum in the environment and in the nutrition of plants, animals and man: Part V: Essentiality and toxicity of molybdenum. Acta Aliment..

[75] Etchegaray A., Haffner D., Cruz S.M., Ogunleye O., Xia J., Schlegel A., Olutoye O.O., Chaudhari B.P. (2025). Early Neonatal Fosdenopterin Treatment for Molybdenum Cofactor Deficiency Type A: New Insights into Its Natural History and Potential Role for Fetal Therapy. J. Clin. Med..

[76] Pu W., Chu X., Guo H., Huang G., Cui T., Huang B., Dai X., Zhang C. (2023). The activated ATM/AMPK/mTOR axis promotes autophagy in response to oxidative stress-mediated DNA damage co-induced by molybdenum and cadmium in duck testes. Environ. Pollut..

[77] Brugman B.L., Scharrer M., Geraci T.S., Navrotsky A. (2023). Cobalt Blues: An Overview of the Thermodynamics of a Critical Element in Short Supply. Mater. Today Energy.

[78] Batyrova G. (2024). The Role of Cobalt in Human Health: A Brief Overview. West Kazakhstan Med. J..

[79] Abed M.S., Moosa A.A., Alzuhairi M.A. (2024). Heavy Metals in Cosmetics and Tattoos: A Review of Historical Background, Health Impact, and Regulatory Limits. J. Hazard. Mater. Adv..

[80] Gregorowicz W., Pajchel L. (2025). The Role of Cobalt Ions in Angiogenesis—A Review. Int. J. Mol. Sci..

[81] Bin Abdulrahman K.A., Alshehri A.F., Almutairi F.M., Almahyawi F.A., Alzahrani R.S., Al-Ahmari O.T., Alshammari K., Aljuhani T.A., Alkadi Y.Y., Alisi M.A. (2025). Assessing the Neurological Impact of Vitamin B12 Deficiency among the Population of Riyadh, Saudi Arabia. Front. Nutr..

[82] Gessner B.D., Steck T., Woelber E., Tower S.S. (2019). A Systematic Review of Systemic Cobaltism After Wear or Corrosion of Chrome–Cobalt Hip Implants. J. Patient Saf..

[83] Angelé-Martínez C., Murray J., Stewart P.A., Haines J., Gaertner A.A.E., Brumaghim J.L. (2023). Cobalt-Mediated Oxidative DNA Damage and Its Prevention by Polyphenol Antioxidants. J. Inorg. Biochem..

[84] Barceloux D.G. (1999). Nickel. J. Toxicol. Clin. Toxicol..

[85] Genchi G., Carocci A., Lauria G., Sinicropi M.S., Catalano A. (2020). Nickel: Human Health and Environmental Toxicology. Int. J. Environ. Res. Public Health.

[86] Schrenk D., Bignami M., Bodin L., Chipman J.K., Del Mazo J., Grasl-Kraupp B., Hogstrand C., Hoogenboom L., Leblanc J., EFSA Panel on Contaminants in the Food Chain (CONTAM) (2020). Update of the risk assessment of nickel in food and drinking water. EFSA J..

[87] Begum W., Rai S., Banerjee S., Bhattacharjee S., Mondal M.H., Bhattarai A., Saha B. (2022). A comprehensive review on the sources, essentiality and toxicological profile of nickel. RSC Adv..

[88] Cempel M., Nikel G. (2006). Nickel: A review of its sources and environmental toxicology. Pol. J. Environ. Stud..

[89] Das K., Reddy R., Bagoji I., Das S., Bagali S., Mullur L., Khodnapur J., Biradar M. (2019). Primary concept of nickel toxicity—An overview. J. Basic Clin. Physiol. Pharmacol..

[90] Yokoi K., Uthus E.O., Nielsen F.H. (2002). The essential use of nickel affects physiological functions regulated by the cyclic-GMP signal transduction system. Proceedings of the 7th International Symposium on Metal Ions in Biology and Medicine.

[91] Kumar A., Trivedi A.V. (2016). A review on role of nickel in the biological system. Int. J. Curr. Microbiol. Appl. Sci..

[92] International Agency for Research on Cancer (IARC) (1990). IARC Monographs on the Evaluation of Carcinogenic Risks to Humans.

[93] Hernberg S. (2000). Lead poisoning in a historical perspective. Am. J. Ind. Med..

[94] Generalova A., Davidova S., Satchanska G. (2025). The Mechanisms of Lead Toxicity in Living Organisms. J. Xenobiotics.

[95] World Health Organization (2021). WHO Guideline for the Clinical Management of Exposure to Lead.

[96] Wani A.L., Ara A., Usmani J.A. (2015). Lead toxicity: A review. Interdiscip. Toxicol..

[97] Raj K., Das A.P. (2023). Lead pollution: Impact on environment and human health and approach for a sustainable solution. Environ. Chem. Ecotoxicol..

[98] Ali S., Naseer S., Rehman M., Wei Z. (2024). Recent trends and sources of lead toxicity: A review of state-of-the-art nano-remediation strategies. J. Nanopart. Res..

[99] Gonzalez-Villalva A., Marcela R.L., Nelly L.-V., Patricia B.-N., Guadalupe M.-R., Brenda C.-T., Eugenia C.-V.M., Martha U.-C., Isabel G.-P., Fortoul T.I. (2025). Lead systemic toxicity: A persistent problem for health. Toxicology.

[100] Larsen B., Sánchez-Triana E. (2023). Global health burden and cost of lead exposure in children and adults: A health impact and economic modelling analysis. Lancet Planet. Health.

[101] Samarghandian S., Shirazi F.M., Saeedi F., Roshanravan B., Pourbagher-Shahri A.M., Khorasani E.Y., Farkhondeh T., Aaseth J.O., Abdollahi M., Mehrpour O. (2021). A systematic review of clinical and laboratory findings of lead poisoning: Lessons from case reports. Toxicol. Appl. Pharmacol..

[102] Charkiewicz A.E., Omeljaniuk W.J., Nowak K., Garley M., Nikliński J. (2023). Cadmium toxicity and health effects—A brief summary. Molecules.

[103] Saini S., Dhania G., Bharagava R., Saxena G. (2020). Cadmium as an Environmental Pollutant: Ecotoxicological Effects, Health Hazards, and Bioremediation Approaches for Its Detoxification from Contaminated Sites. Bioremediation of Industrial Waste for Environmental Safety.

[104] Suhani I., Sahab S., Srivastava V., Singh R.P. (2021). Impact of cadmium pollution on food safety and human health. Curr. Opin. Toxicol..

[105] Nordberg G.F., Bernard A., Diamond G.L., Duffus J.H., Illing P., Nordberg M., Skerfving S. (2018). Risk assessment of effects of cadmium on human health (IUPAC Technical Report). Pure Appl. Chem..

[106] Vijiyakumar N., Prince S.E. (2025). A comprehensive review of cadmium-induced toxicity, signalling pathways, and potential mitigation strategies. Toxicol. Environ. Health Sci..

[107] Genchi G., Sinicropi M.S., Lauria G., Carocci A., Catalano A. (2020). The effects of cadmium toxicity. Int. J. Environ. Res. Public Health.

[108] Davidova S., Milushev V., Satchanska G. (2024). The mechanisms of cadmium toxicity in living organisms. Toxics.

[109] Kuna G., Gullipalli S., Chintada V. (2024). Health risks associated with cadmium toxicity. Cadmium Toxicity in Water: Challenges and Solutions.

[110] Del Carpio E., Hernández L., Ciangherotti C., Villalobos-Coa V., Jiménez L., Lubes V., Lubes G. (2018). Vanadium: History, chemistry, interactions with α-amino acids and potential therapeutic applications. Coord. Chem. Rev..

[111] Rehder D. (2013). Vanadium. Its role for humans. Met. Ions Life Sci..

[112] Pessoa J.C., Etcheverry S., Gambino D. (2015). Vanadium compounds in medicine. Coord. Chem. Rev..

[113] Rojas-Lemus M., López-Valdez N., Bizarro-Nevares P., González-Villalva A., Ustarroz-Cano M., Zepeda-Rodríguez A., Pasos-Nájera F., García-Peláez I., Rivera-Fernández N., Fortoul T.I. (2021). Toxic Effects of Inhaled Vanadium Attached to Particulate Matter: A Literature Review. Int. J. Environ. Res. Public Health.

[114] Mukherjee B., Patra B., Mahapatra S., Banerjee P., Tiwari A., Chatterjee M. (2004). Vanadium—An element of atypical biological significance. Toxicol. Lett..

[115] Ścibior A., Llopis J., Holder A.A., Altamirano-Lozano M. (2016). Vanadium Toxicological Potential versus Its Pharmacological Activity: New Developments and Research. Oxidative Med. Cell. Longev..

[116] Tripathi D., Mani V., Pal R.P. (2018). Vanadium in Biosphere and Its Role in Biological Processes. Biol. Trace Elem. Res..

[117] Panchal S.K., Wanyonyi S., Brown L. (2017). Selenium, Vanadium, and Chromium as Micronutrients to Improve Metabolic Syndrome. Curr. Hypertens. Rep..

[118] Rehder D. (2015). The role of vanadium in biology. Metallomics.

[119] Fortoul T.I., Rodriguez-Lara V., Gonzalez-Villalva A., Rojas-Lemus M., Cano-Gutierrez G., Ustarroz-Cano M., Colin-Barenque L., Montaño L.F., García-Pelez I., Bizarro-Nevares P. (2011). Vanadium inhalation in a mouse model for the understanding of air-suspended particle systemic repercussion. J. Biomed. Biotechnol..

[120] Ścibior A., Pietrzyk Ł., Plewa Z., Skiba A. (2020). Vanadium: Risks and possible benefits in the light of a comprehensive overview of its pharmacotoxicological mechanisms and multi-applications with a summary of further research trends. J. Trace Elem. Med. Biol..

[121] López-Valdez N., Gonzalez-Villalva A., Rojas-Lemus M., Bizarro-Nevares P., Casarrubias-Tabarez B., Cervantes-Valencia M.E., Ustarroz-Cano M., Guerrero-Palomo G., Morales-Ricardes G., Salgado-Hernández J.Á. (2025). Vanadium, a Promising Element for Cancer Treatment. Inorganics.

[122] DesMarais T.L., Costa M. (2019). Mechanisms of Chromium-Induced Toxicity. Curr. Opin. Toxicol..

